# Feasibility, acceptability and effectiveness of digitally delivered multimodal prehabilitation for cancer patients: A mixed-methods systematic review

**DOI:** 10.1371/journal.pdig.0001258

**Published:** 2026-03-02

**Authors:** Jeremiah Oyedemi, Simon Dunne, Louise Brennan, Laura Coffey

**Affiliations:** 1 Department of Psychology, Maynooth University, Maynooth, Co. Kildare, Ireland; 2 School of Psychology, Dublin City University, Glasnevin, Dublin, Ireland; 3 Mercer’s Institute for Successful Ageing, St James’s Hospital, Dublin, Ireland; 4 Discipline of Physiotherapy, School of Medicine, Trinty College Dublin, Dublin, Ireland; Polytechnic Institute of Porto: Instituto Politecnico do Porto, PORTUGAL

## Abstract

Multimodal prehabilitation, which encompasses physical, nutritional, and psychological strategies, is increasingly recognised for its potential to prepare cancer patients holistically for their treatment journey. This mixed-methods systematic review assessed the feasibility, acceptability, and effectiveness of digitally delivered multimodal prehabilitation interventions for cancer patients. A systematic search was conducted in Embase, PubMed, Scopus, PsycINFO, and Web of Science for studies published from January 2008 onwards. Eligible studies included quantitative, qualitative, and mixed-methods designs reporting on digital multimodal prehabilitation interventions (encompassing physical, nutritional, and psychological components) for adult cancer patients in the pre-treatment phase. Screening, data extraction, and quality appraisal were performed independently by two reviewers, with disagreements resolved through discussion. Data were synthesized narratively using a convergent segregated approach. Twelve studies (n = 602 participants) met the inclusion criteria, with most focusing on gastrointestinal, gynaecological, urologic/genitourinary, and breast cancer. Most interventions utilized wearable fitness trackers, telehealth platforms, alongside mobile health applications to deliver prehabilitation. Feasibility and acceptability were generally high, with positive patient feedback and good adherence. Consistent improvements in physical function (e.g., walking capacity, strength) were observed across studies, but psychological outcomes were mixed and nutritional status was underreported. Five studies (qualitative) highlighted patient satisfaction but noted barriers such as digital literacy. Limited evidence suggested possible reductions in hospital stays and complications. The findings suggest that digitally delivered multimodal prehabilitation is feasible and can enhance physical preparedness for cancer treatment, with high patient satisfaction. However, standardization of interventions and further research on psychological and nutritional outcomes are needed to optimize effectiveness and ensure equitable access across diverse cancer populations. Future research should focus on diverse cancer populations and use co-design to ensure interventions are tailored to their needs.

## Author summary

In recent years, digital health interventions have shown promise in supporting cancer patients before treatment. In this review, we examined studies delivering multimodal prehabilitation, combining exercise, nutrition, and psychological support, through digital platforms. We found that these interventions often achieved high patient engagement, especially when content was personalised and easily accessible. However, some participants expressed dissatisfaction with psychological components, highlighting the need for user-friendly design across all programme aspects. Notably, only few of the studies we reviewed involved patients or healthcare professionals in co-designing these programmes, a step we believe is crucial for improving their usefulness and appeal. While most participants who started these programmes maintained adherence, technology issues and varying digital literacy led to some dropouts. Our review is the first to examine digital, multimodal prehabilitation in cancer care through a comprehensive, multidisciplinary lens. We conclude that while digital prehabilitation shows promise for improving patient outcomes and reducing hospital stays, future efforts should focus on standardising programme features, including diverse patient populations, and ensuring equitable access to these innovations.

## Introduction

In cancer care, the trajectory from diagnosis through treatment and beyond has traditionally focused on therapeutic interventions aimed at disease eradication and symptom management. However, there is growing evidence for the role of prehabilitation in cancer care [1]. Prehabilitation is defined as preparatory health optimisation before beginning cancer treatments such as surgery, chemotherapy, or radiation [2]. Most studies to date have focused on exercise prehabilitation, i.e., a unimodal prehabilitation programme, but recent research suggests that combining exercise with other aspects of pre-treatment care, such as nutrition and psychological supports, could yield substantial improvements in patient outcomes [3,4]. The move towards multimodal prehabilitation recognises that physical, nutritional, and mental health are all connected and can help people be more resilient and recover faster after treatment [5,6]. This integration of multidisciplinary interventions not only enhances the effectiveness of individual components but also supports a more holistic patient experience.

The integration of digital technologies into healthcare has opened new avenues for delivering and enhancing cancer prehabilitation [7]. Digital health tools, including telehealth platforms, wearable fitness devices and mobile health applications, have emerged as pivotal elements in administering continuous, personalised care to cancer patients [8,9]. These technologies facilitate remote monitoring and management of patient health, allowing for tailored interventions that adapt to the changing needs of patients throughout their cancer journey. Digital tools offer the unique advantage of engaging patients in their care process from the comfort of their own homes, ensuring they receive necessary guidance and support without the need for frequent hospital visits [10]. For prehabilitation, home-based, digitally enabled care has the potential to increase programme reach and scalability, as well as enhance patient adherence and satisfaction, factors that are crucial for the success of such interventions [11,12]. Leveraging digital tools in multimodal prehabilitation combining physical exercise, nutritional optimisation and psychological support could allow for a seamless integration of these components, providing a comprehensive, customisable pre-treatment plan that addresses the multifaceted needs of patients [13,14].

Multimodal digital prehabilitation interventions are typically delivered through live or pre-recorded video sessions, educational modules, or telehealth appointments enabled by mobile applications or web-based platforms, often enhanced by personal health data from wearable devices [15–19]. This approach simultaneously optimises physical fitness, nutritional status, and mental health prior to cancer treatment through remotely delivered, personalised exercise regimens, diet management plans, and psychological support [18]. Early feasibility studies indicate that cancer survivors undergoing digital multimodal prehabilitation exhibit better physical fitness, nutritional status, and enhanced psychological readiness [20], which collectively contribute to improved treatment outcomes [21], quality of life (QoL) [7], and potentially reduced recovery times post-treatment [22].

While early feasibility studies on multimodal digital prehabilitation show promising results [23–26], there is a lack of aggregated data on their feasibility, acceptability and effectiveness among cancer survivors, making it difficult to draw definitive conclusions about its potential value and impact in routine cancer care. While individual studies may offer valuable insights into specific populations or settings, a synthesised overview capturing broader trends in the perceived utility, satisfaction, and acceptance of such interventions among patients, as well as their effectiveness in improving patient outcomes, is needed [24–26]. The aim of the present systematic review was thus to systematically synthesise the published evidence on the feasibility, acceptability and effectiveness of digitally delivered multimodal prehabilitation interventions for cancer patients. The specific objectives were threefold: 1) to evaluate the feasibility and acceptability of multimodal digital prehabilitation 2) to assess their effectiveness on patient outcomes such as physical fitness, psychological readiness, nutritional status and 3) to explore patient and provider perspectives on their use in cancer care.

## Method

### Design

A mixed-methods systematic review was conducted following the Joanna Briggs Institute (JBI) methodology [27,28] for conducting mixed-methods systematic reviews and the Preferred Reporting Items for Systematic Reviews and Meta-Analysis (PRISMA) guidelines [29]. The review protocol was registered on PROSPERO in February 2024 (reference number: CRD42024508536).

### Eligibility criteria

To ensure a focused and relevant analysis, the following eligibility criteria were applied:

**Study design**: Quantitative, qualitative, and mixed-methods studies that presented primary data on the use of digital technologies in multimodal prehabilitation for cancer patients were eligible for inclusion.
**Participants**: Studies that included cancer survivors from any demographic or type/stage of cancer and/or healthcare professionals participating in cancer care aged 18 years or above were eligible for inclusion, as long as the focus of the intervention was on the prehabilitation phase before any primary cancer treatment such as surgery, chemotherapy, or radiation.
**Interventions**: Included studies were required to involve a multimodal prehabilitation intervention encompassing physical, nutritional and psychological strategies, delivered using digital technologies such as wearable fitness trackers, telehealth platforms, and mobile health applications.
**Outcomes**: The primary outcomes of interest were feasibility, acceptability and patient outcomes such as physical fitness levels, psychological readiness, nutritional status and QoL.
**Timeframe**: Studies published from January 2008 onwards were considered eligible for inclusion, to reflect recent advancements in digital technology and its applications in healthcare.
**Publication status**: Peer-reviewed articles in English presenting primary data were eligible for inclusion. Conference abstracts, editorials, commentaries, protocols, and reviews were excluded.

### Search strategy

To explore the available literature on the review questions, a pilot search was conducted by JO in Google Scholar, PROSPERO, and other selected databases during the first week of January 2024. The purpose was to further establish the objectives of the review and the eligibility criteria, develop the search strategy, and confirm there were no previous or continuing reviews on the subject. A main search was conducted on February 24, 2024, across five electronic databases: Embase, PubMed, Scopus, PsycINFO, and Web of Science. The search strategy aimed to capture all relevant articles using a combination of controlled vocabulary and free-text terms related to the keyword’s “cancer”, “prehabilitation” and “digital technologies”. The reference lists of articles selected for inclusion were manually searched by JO for any additional eligible papers. A supplementary search of Google Scholar using the above keywords was conducted in June 2024 to identify any articles that had been published since the main search. See S1 Appendix for more details on search strategy.

### Article screening

All records identified in the search were imported into Rayyan [30], a collaborative web-based software designed for systematic review management. Duplicates were removed automatically by the software, and a supplementary manual check on the title was conducted by JO in Microsoft Excel to ensure no duplicates were missed. Title/abstract screening was carried out independently by two reviewers (JO and LC). Full texts of the remaining articles were reviewed independently for eligibility by JO and LC; any disagreements between the reviewers were resolved through discussion. Studies deemed eligible for inclusion underwent data extraction and synthesis. Outcome of article screening can be found in S2 Appendix.

### Data extraction

Standard data extraction forms were developed by JO to capture all relevant data required to address the review’s objectives, including study characteristics, participant demographics, outcomes, and key findings. Intervention details were also captured using a modified Template for Intervention Description and Replication (TIDieR) checklist (see S3 Appendix). The forms were pilot tested by JO on a small sample of included studies and reviewed by LC to ensure they adequately captured all necessary information. The data extracted from each study included:

**Study details and participant characteristics**: Author(s), year of publication, country in which study was conducted, study design, sampling methods, sample size, age, gender distribution, cancer type.
**Interventions**: Type of intervention, components (physical, nutritional, psychological), duration/frequency, delivery mode/technology.
**Outcome:** Outcomes were split into three main categories: i) implementability or feasibility (e.g., recruitment rate, retention rate, technology feasibility, adherence); ii) acceptability (e.g., user satisfaction and feedback); and iii) patient outcomes (e.g., physical function/activity levels, psychological wellbeing and QoL, length of hospital stay, complications).

JO conducted the data extraction. To ensure the accuracy of the extracted data, a second round of checks was performed by JO randomly on the extracted data, and the final extracted data was reviewed by LC. All the data extraction can be found in S3 Appendix.

### Quality appraisal

The quality of included studies was assessed independently by JO and LC using the Mixed Methods Appraisal Tool [31]. This tool comprises two initial screening questions, followed by five design-specific questions (which differ for qualitative, quantitative randomized controlled trial, quantitative non-randomized, quantitative descriptive or mixed-methods studies). Studies with 4–5 criteria met were interpreted as of high quality, 2–3 criteria met as of moderate quality, and 0–1 criteria met as of low quality. Quality reviews were conducted to aid readers’ critical consideration of the credibility of the included studies’ findings and were not used as a basis for exclusion.

### Data synthesis

A convergent segregated approach to synthesis and integration was employed, in accordance with the JBI methodology for mixed-methods reviews [27]. This involved separate analyses of quantitative and qualitative data, followed by their integration. Due to the limitations of statistical and textual pooling, both sets of findings were presented narratively using content analysis of the extracted data as recommended by the JBI approach, supplemented by tables and figures as necessary. Similarly, as configuration was not feasible, the integration of quantitative and qualitative evidence was also presented in narrative form as suggested by the JBI approach. Data synthesis and integration were conducted by JO and reviewed by LC.

## Results

### Study selection

**Fig 1:**
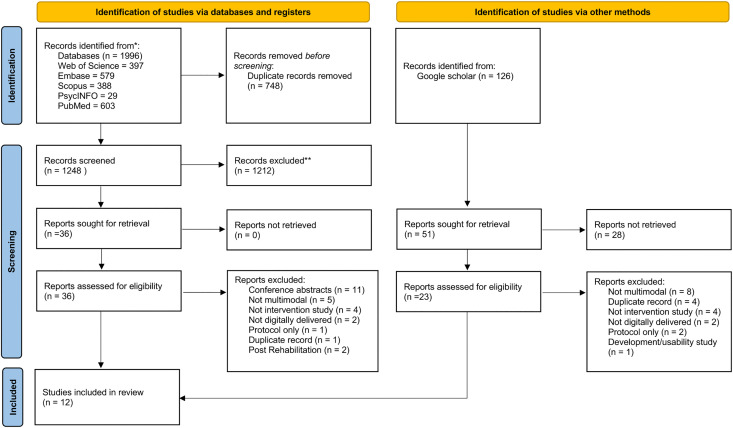
PRISMA flow diagram. Adapted from Page et al. [29].

We screened a total of 1,996 articles by their titles and abstracts and assessed 59 full texts for eligibility. Twelve papers met the eligibility criteria and were included in the review. The results of the database and reference searches are shown in the PRISMA flowchart in Fig 1.

### Study characteristics

Characteristics of the included studies are summarised in Table 1. The study quality was assessed using the Mixed Methods Appraisal Tool (MMAT; detailed in S1 Appendix). MMAT scores ranged from 20% [26] to 100% ([32], with the distribution as follows: one study scored 100% [32], two scored 80% [33,34], three scored 60% [23,35–37], five scored 40% [24,25,38,39], and one scored 20% [26]. Most studies (n = 8) received moderate scores (40–60%) primarily due to having single-arm designs without control groups (MMAT quantitative non-randomised study designs Q4: confounders not accounted for), despite using appropriate outcome measurements. All studies selected for inclusion were conducted in Western countries, including the United Kingdom (*n* = 5), Canada (*n* = 2), Australia (*n* = 2), the Netherlands (*n* = 1), and Ireland (*n* = 1), except for one study [25] originating from China. There was a total of 602 participants across the included studies, with sample sizes ranging from 6 [34] to 192 [39] participants. The mean or median age of participants spanned from 19 to 95 years, with most studies including both male and female participants, though one study focused exclusively on females [25] and another did not specify gender [34]. Gastrointestinal (including colorectal) cancers were the most frequently reported cancer type, being included in 10 studies [23,24,26,32,33,35–39], and accounting for the highest number of cases (n = 329, 54.7%). Gynaecological cancers were reported in three studies [25,35,37], with a total of 101 participants (16.8%). Urologic/genitourinary cancers were included in 5 studies [32,35,37–39], totalling 72 cases (12.0%). Breast cancer appeared in five studies [32,35,37–39], accounting for 53 cases (8.8%). Other cancer types had lower representation: lung cancer in three studies [37–39] with 11 cases (1.6%), head and neck cancers in three studies [35,37,39] with 8 cases (1.3%), hepatobiliary cancers in two studies [35,38].

**Table 1:** Characteristics of included papers.

Authors/year	Country/location	Study design	Sample Size	Age	Sex	Aim of the study	Type of cancer	Quality Assessment score
Bennett et al., 2023	Ireland	Pilot implementation/ quality improvement initiative	6	Range: 43–70 years old	Gender not specified	To evaluate the implementation of a radiation therapist-led oncology prehabilitation programme that provides psychological and physical support to patients before starting cancer treatment	Prostate, Breast, Colorectal	4
Bruns et al., 2018	Netherlands	Pilot feasibility study	14	Median age: 79 years; range: 74–86 years	Female: 9 (64%); male: 5 (36%)	To assess the feasibility of Fit4SurgeryTV, an at-home prehabilitation programme specifically designed for frail elderly with colorectal cancer	Colorectal	4
Gkaintatzi et al., 2022	United Kingdom	Observational cost analysis study	192	Male: mean age 66.92 years, female: mean age 61.77 years	Female: 74 (38.5%); male: 118 (61.5%)	To assess the impact on health-related quality of life (HRQoL) and the costs of a digital multimodal prehabilitation programme	Colorectal, Urologic, Breast, Lung, Brain, Head and neck, Pancreatic, Prostate	2
Li et al., 2024	China	Non-concurrent control study	97 (48 control + 49 intervention)	Mean ± SD: control 54.9 ± 8.7 years; intervention 54.1 ± 9.9 years	Female: 97 (100%)	To explore the effect of a short-term, hospital-based, multimodal preoperative prehabilitation intervention on perioperative functional ability of patients with gynecological malignant tumours	Cervical, Endometrial, Ovarian	2
MacDonald et al., 2020	Canada	Mixed methods pilot study	35	Mean age: 55 years; range: 19–76 years	Female: 22 (62.9%); male: 13 (37.1%)	To assess the feasibility, acceptability, and preliminary impact of the CaRE@Home programme, an 8-week virtual cancer rehabilitation and exercise initiative designed to restore and optimize function and well-being in individuals with cancer-related impairments	Breast, Gastrointestinal, Lymphoma and myeloma, Gynecological, Endocrine, Head and neck, Central nervous system, Genitourinary, Leukemia, Lung	3
Moorthy et al., 2023	United Kingdom	Feasibility/ implementation comparative study	57 (31 digital + 26 in-person)	Mean age: 67.4 (8.9) years for digital, 65 (10.1) years for in-person	Female: 14 (25%); male: 43 (75%)	To establish the feasibility of delivering a digital prehabilitation service	Oesophageal, Gastric, Gastro-oesophageal junction	1
Piche et al., 2023	Canada	Prospective single-group pragmatic feasibility study	25	Mean ± SD 60.2 ± 14.0; range 24–78 years	Female: 20 (80%); male: 5 (20%)	To develop a group-based, multimodal, tele-prehabilitation intervention for individuals diagnosed with cancer and assess its implementability in a “real-world” clinical setting by measuring feasibility, acceptability, fidelity, and preliminary effects	Breast, Prostate, Liver, Colorectal, Bladder, Oral, Vulva	3
Steffens et al., 2023	Australia	Cross-sectional study	30	Median: 54 years; range: 23–95 years	Female: 24 (80%); male: 6 (20%)	To explore patients’ perspectives on the adoption of a prehabilitation multimodal online programme	Gastrointestinal	2
Waller et al., 2022	United Kingdom	Randomised controlled pilot study (RCT)	22 (11 intervention + 11 control)	Mean age: 55.5 years (prehab) and 61.0 years (control)	Female: 11 (50%); male: 11 (50%)	To assess the efficacy of a trimodal prehabilitation programme delivered by smartwatches for improving functional fitness prior to major abdominal cancer surgery	Colorectal adenocarcinoma, Pseudomyxoma peritonei, Other	3
Waterland et al., 2021	Australia	Implementation/ impact evaluation	35	Mean age: 59 years	Female: 19 (54%); male: 16 (46%)	To evaluate the current and likely future impact of a telehealth preoperative education package for patients preparing for major abdominal cancer surgery	Abdominal	3
Wu et al., 2021	United Kingdom	Prospective, cohort observational study (service evaluation)	66	Mean age: 67 years; range: 60–73 years	Female: 32 (48%); male: 34 (52%)	To establish the feasibility and effects of a telehealth-delivered home-based prehabilitation programme during the COVID-19 pandemic	Colorectal, Urological, Breast, Lung, Hepatobiliary, Brain, Unknown	2
Wu et al., 2022	United Kingdom	Descriptive qualitative study with semistructured interviews	22	Median age: 66 years; range: 42–83 years	Female: 11 (50%); male: 11 (50%)	To qualitatively explore patients’ experiences and perspectives of tele-prehabilitation using COM-B model of behaviour change, to inform service redesign and delivery	Colorectal, Breast, Urological	5

**Note:** Quality assessment score represents the number of Mixed Methods Appraisal Tool (MMAT) criteria met out of 5 (range: 1–5; representing 20%–100%). Study design-specific criteria were applied for non-randomised studies, qualitative studies, mixed methods studies, and randomised controlled trials. Full quality appraisal details are provided in S1 Appendix.

### Intervention characteristics

Interventions characteristics are summarised in Table 2. Most studies (n = 11) involved interventions that were delivered remotely, with only one study involving a hospital-based digital intervention [25]. Eleven studies involved full implementation of the interventions, two of which focused on the same telerehabilitation programme [33,38]. The remaining study [24] explored the views and expected outcomes of a prehabilitation multimodal online programme among patients recovering from gastrointestinal cancer surgery, who had access to the intervention for a 24-hour period [33,38]. The digital platforms employed varied considerably across studies. Three studies utilised synchronous videoconferencing via Zoom as their primary digital platform [23,34,35]. Two studies [38,39] involved using telephone and video calls through telehealth services. Custom mobile applications featured prominently, with three studies implementing interventions through mobile applications with wearable technology integration, specifically Fitbit devices [26,36,37]. Three other studies employed standalone mobile applications [24,33,39]. One study employed social media (WeChat) and fitness applications (Keep APP) [25].

**Table 2:** Characteristics of interventions in included papers.

Study Reference	Total Duration of Intervention	Digital Technology	Exercise Intervention Summary	Nutrition Intervention Summary	Psychology Intervention Summary
Bennett et al. (2024)	4-6 weeks; 1hr sessions twice/week	STRONG programme (Supporting Prehabilitation in Oncology) on Zoom platform	Personalised supervised exercise sessions focusing on cardiovascular and strengthening exercises using a Theraband, conducted weekly over Zoom	Nutritional advice through presentations and access to online resources, focusing on general education about nutrition for cancer patients	Psychological support included sessions on practical and psychosocial challenges, alternating weekly with exercise sessions
Bruns et al. (2018)	Median 26 days (range 18–32 days)	Fit4SurgeryTV digital device	Daily elderly-adapted strength training based on an adapted seven-minute workout; device provides prompts/instructions	Generic protein-rich diet plan featuring two daily meals (breakfast and snack, 20-30g protein each)	Generic social reward system delivered providing digital awards for completed exercises/recipes with a final reward of zoo passes upon program completion
Gkaintatzi et al. (2022)	<3 to>10.5 weeks	Craetus platform (apps and emails) with remote consultations	Personalised activity via craetus platform based on functional capacity	Personalised nutritional guidance based on remote consultations assessing patient needs	Personalized psychological support
Li et al. (2024)	Daily from day of admission to 30th day after surgery	WeChat & Keep APP	Individualized: warm-up (5 min), resistance (10 min alternate days), aerobic, respiratory (5 min daily), Kegel (10 min before bed)	Personalised diet plan with specific carbohydrate:protein:fat ratio (6:3:1) and protein intake (1.2g/kg/day)	Yoga relaxation techniques, deep abdominal breathing, muscle relaxation, and meditation for ~20 min daily before lunch
MacDonald et al. (2020)	8-week programme; 6 health coaching calls during weeks 2–7, 8 weekly e-modules	Physitrack app/ Fitbit/e-learning modules/telephone health coaching	Individualized progressive aerobic/strength/flexibility exercises via app with Fitbit tracking	Nutritional education via weekly e-modules	Personalised weekly health coaching calls (6 calls during weeks 2–7) informed by motivational interviewing, e-modules for self-management skills
Moorthy et al. (2023)	Median 12 weeks	Onkohealth app, Fitbit Inspire 2 device	Personalised aerobic & strength plans via app with Fitbit tracking	Personalised nutritional behaviour change guidance focusing on quality and eating behaviours	Personalised psychological wellbeing support addressing emotional wellbeing and self-efficacy
Piché et al. (2023)	Median 28 days (based on surgical wait time)	Live Zoom sessions in groups of 2–10 participants	Semi-personalised exercise programme circuit training via live Zoom sessions 3x/week (60 min)	Generic nutrition education delivered through interactive discussions during 30-minute educational sessions three times weekly	Generic psychological support delivered through interactive educational sessions in 30-min sessions
Steffens et al. (2023)	Evaluation phase only - actual programme duration not implemented 24-hour access to the intervention	iPad e-Health app (StandingTall)	Generic functional balance & strength exercises via app (including warm-up, sit-to-stand, and stepping exercises)	Generic nutritional advice via app	Pre-recorded psychological advice via app
Waller et al. (2022)	Variable based on time from enrollment to surgery (approximately 4–5 weeks). Mean duration from enrolment to day before surgery was 30.5 days	Fitbit Charge 2 & Smiling Mind app	Semi-Personalised structured aerobic 3x/week, resistance 2x/week, additional 30-min physical activity on 2 other days; guided by Karvonen formula and BORG Scale	Generic dietary advice on avoiding weight loss and increasing protein consumption; monitored via Fitbit App food log	Pre-recorded mindfulness practice through daily guided meditation, delivered through mindfulness app (Smiling Mind) with interactive weekly phone support
Waterland et al. (2021)	Delivered 7 times over 4-month period, each session lasted 75 minutes, offered bimonthly	Online webinar, video repository (Vimeo) and presentation	Pre-recorded presentations on exercise: avoiding inactivity, meeting physical activity guidelines, performing breathing exercises 5–10 min daily via webinar with ongoing access	Pre-recorded presentations on nutrition: maintaining weight, increasing muscle through protein, following healthy eating guidelines	Pre-recorded psychological support included education on psychological preparedness and relaxation, strategies to manage worry (e.g., relaxation techniques), motivation and goal setting
Wu et al. (2021)	Referral until one week before surgery or throughout non-surgical cancer treatment	Telehealth & online videos	Personalised exercise program including moderate intensity physical activity, resistance exercises, and specialized exercises for specific surgeries	Personalised nutritional education emphasising non-processed foods and protein intake (1.5g/kg)	Personalised individual counselling

All interventions incorporated exercise, nutritional and psychological components, with significant variance in personalisation approaches. Regarding exercise, six studies implemented personalised exercise programmes tailored to individual patient needs [25,26,34,37–39]. In contrast, four studies employed standardised, generic exercise protocols [24,33,35,36], while one study [23] provided only general activity guidance. Exercise delivery methods included pre-recorded instructions [23,24,33,36,38] and interactive professional guidance [25,26,34,35,39]. Nutritional components showed similar variation in personalisation. Five studies implemented personalised nutritional guidance based on individual assessment [25,26,34,38,39], typically involving dietary analysis, specific nutritional targets and ongoing adjustments. The remaining six studies provided generic nutritional education [23,24,33,35–37]. A notable pattern emerged in protein recommendations, with some studies [23,33,38] explicitly specifying protein intake targets. Personalised psychological interventions featured in six studies [25,26,34,37–39], typically delivered through individual or group counselling sessions. Generic psychological support was implemented in the other studies, often through standardised content such as mindfulness applications or pre-recorded sessions. One study [24] used a unique design, recruiting post-operative patients to navigate and evaluate a prehabilitation app over a 24-hour period to assess anticipated barriers and facilitators to adoption. Whilst this differed from other implementation studies included in the review that delivered interventions pre-operatively, it was included under patient perspectives as it provided consumer insights on prehabilitation programme acceptability and anticipated feasibility challenges.

### Feasibility of multimodal digital prehabilitation

#### Recruitment and dropout rates.

Recruitment rates across studies varied considerably, with three studies exceeding 75% [24,26,32,38], and two studies achieving a 100% recruitment rate [24,32]. Four studies demonstrated moderate recruitment rates of 50–74% [23,34,36,37], while one study [35] had the lowest recruitment rate of 29%. Dropout rates were reported in 10 of the included studies [23,25,26,32–38] and ranged from 0% to 33%. Two studies [33,36] achieved remarkable success in maintaining participant involvement, with dropout rates of 0%. In contrast, other studies noted some dropouts and highlighted different challenges in participant retention. Bennett et al. [34] reported the highest dropout rate at 33%, where participants were lost due to treatment-related fatigue and earlier-than-anticipated surgical resection. In Moorthy et al.’s study [26], both the digital and in-person groups had a dropout rate of 10%.

#### Adherence.

Adherence rates were reported in all studies and ranged from 70.2% to 100%. Despite some participants facing issues like treatment-related fatigue, Bennett et al. [34] observed a 97% adherence rate overall, while Li et al. [25] achieved 100% adherence in their intervention group. Moorthy et al. [26] reported 86% adherence for their digital programmes, significantly higher than the 71% adherence rate observed for in-person programmes. Conversely, Waterland et al. [23] faced issues with participants not attending scheduled online sessions, with some not contactable for follow-up. Waller et al. [36] reported that prehabilitation group participants wore their Fitbit devices on 98.9% of days. Engagement with the mindfulness app was notably lower, however, with usage at only 15% of days, impacted by participants’ perceptions of their baseline mental health and the app’s usefulness. MacDonald et al. [37] reported varying levels of adherence within different components of their programme; while the health coaching intervention saw around 80% of participants completing at least five calls, module completion rates were slightly lower at 77%, with a significant drop-off for later modules. Only Bruns et al. [33] explicitly defined parameters for successful adherence, noting that previous research on adherence to at-home lifestyle interventions in other patient groups has shown a wide range of compliance rates (16–67%) and defined success as when an average of 70% adherence is obtained. Using this 70% benchmark, most studies in this review achieved successful adherence rates, suggesting that telehealth and digital prehabilitation interventions can achieve compliance levels comparable to traditional in-person programmes despite the unique challenges of remote delivery.

#### Technology feasibility.

The usability of the various technologies employed in the included interventions was rated quite highly by participants for the most part. In four studies, the user interfaces of the digital health tools included in the interventions were found to be very clear and easy to use [23,24,33,34]. Bennett et al. [34] reported that most participants found using digital tools like Zoom easy, enhancing programme accessibility. Similarly, Bruns et al. [33] noted that 86% of users rated the user interface of their mobile application as clear. Waterland et al. [23] found that their mobile application was easy to use for 97% of participants; 23% of them used the content repeatedly, indicating good usability and user engagement. Lastly, Steffens et al. [24] reported that 92% of their participants found the online programme instructions easy to understand. Some technological challenges were reported across studies, such as difficulties with gadgets, technical issues, and lack of confidence with mobile devices [23,24,26,33,38]. For instance, 14% of participants in Bruns et al.‘s [33] study had difficulties with the touchscreen of their mobile devices; similarly, Steffens et al. [24] noted that some participants reported a lack of confidence using mobile devices. Participants in Moorthy et al.’s study [26] faced substantial initial challenges with wearable device connectivity, with only 25% of participants’ wearable data collected in the first half of the study, although this improved to 86% in the second half. In other studies, participants reported requiring technical support. For example, Steffens et al. [24] found that 21.4% of participants needed technical support to successfully navigate the programme, 6.9% of whom reported feeling unconfident when using the programme. Waterland et al. [23] also noted that although their intervention was predominantly user-friendly, 14% of participants had to seek assistance from family or friends to configure their sessions. Another participant had to access the intervention from a local community centre with the help of centre staff, as they did not have the required technology at home.

### Acceptability of multimodal digital prehabilitation

#### Feedback and participant satisfaction.

Interventions were generally positively received, with participants across 9 studies [23,25,33–39] reporting high levels of overall satisfaction. In Bennett et al.’s study [34], for example, all participants perceived their programme as a positive quality improvement initiative, and were satisfied with features such as privacy protection, knowledge acquisition, and reduced feelings of isolation. Similarly, Bruns et al. [33] reported an overall participant satisfaction median grade of 8 out of 10, indicating high approval. High satisfaction with digital platforms was also evident in Li et al.’s study [25] where participants reported a median usability score of 85, signifying good user-friendliness; Steffens et al. [24] reported similarly high satisfaction scores of 4.5 out of 5 for their mobile application. Participants in studies by Gkaintatzi et al. [39] and Wu et al. [38] also expressed high overall satisfaction with their digital prehabilitation programmes, praising their effectiveness and user-friendliness. Participants across two studies reported feeling well-prepared for surgery due to their programmes, with 88% in Piché et al.’s [35] study feeling well-prepared.

Participants across 5 studies [25,33–36] reported positive feedback on individual components of their prehabilitation programmes. The exercise component was often highly rated, with 100% of participants in Waller et al.’s [36] study rating both the overall programme and the exercise component as “good” or “excellent” and about 90% agreeing that the Fitbit motivated them to perform physical activity. Piché et al. [35] also reported 100% participant satisfaction with their programme’s exercise component and supervision by kinesiologists. Similarly, Bruns et al. [33] reported that patients specifically appreciated the at-home exercises, and 64% reported perceived physical improvement. Participants in Li et al.’s study [25] also valued individualised exercise plans and reported feeling more prepared for surgery as a result. In Bennett et al.’s study [34], all participants ranked exercise and diet as the most valuable components of their interventions, appreciating the protection of privacy, gaining knowledge, and feeling less isolated through their programmes. Positive feedback on the dietary components of interventions was also evident, with 92% of participants in Piché et al.’s study [35] reporting learning useful nutritional information from their programme. Likewise, over 70% of participants in Steffen et al.’s study [24] agreed that the nutritional information they were provided with was relevant to them. Positive feedback was also noted regarding remote consultations and the comprehensive support provided by some programmes [23,38]. Participants in Waterland et al.’s study [23] appreciated the convenience of not having to travel and valued being able to involve family members in their online sessions. Approximately 77% of participants also preferred online over hospital-based sessions. Wu et al.’s [38] participants reported that telehealth delivery prevented the need to exercise publicly, offered flexibility around medical appointments, and provided continuous social support.

Participants across four studies identified some areas for improvement within the programmes, including more flexibility in module completion and remote options for in-person assessments [37]. In Piché et al.‘s [35] study, some participants preferred morning sessions over the scheduled afternoon ones. Bruns et al. [33] mentioned that a small proportion of participants found the recipes in their nutritional intervention too difficult (1.7%) or not tasty (4.3%). Feedback on the psychological component of interventions was limited, although Waller et al. [36] noted that feedback on the mental wellbeing component of their programme was mixed, with some participants finding the mindfulness app unhelpful.

### Health-related outcomes of multimodal digital prehabilitation

#### Psychological and physical activity outcomes in multimodal digital prehabilitation interventions.

Psychological responses to multimodal digital prehabilitation interventions demonstrated notable variability across studies. While some interventions yielded significant improvements, others showed minimal or non-significant psychological benefits, as seen in Table 3. In three studies, depression and anxiety measurements frequently showed modest improvements that failed to reach statistical significance [26,35,37]. Quality of life metrics also demonstrated inconsistent patterns, with significant improvements in some studies [33,39], while others showed slight non-significant increases [37] or even decreases in global health status [35]. The clearest contrast in psychological outcomes emerged when examining studies with control group designs. A hospital-based digital multimodal prehabilitation intervention [25] demonstrated significant between-group differences in psychological parameters, with participants showing significantly reduced anxiety before surgery compared to controls. This advantage was maintained at 30 days post-surgery, and participants also exhibited significantly higher recovery quality scores across multiple post-surgical time points. In contrast, a wearable-based intervention [36] showed no significant between-group differences in anxiety or depression measurements despite the same intervention producing significant improvements in physical parameters (Tables 3 and 4).

**Table 3:** Psychological outcomes and results reported in included paper.

Study Reference	Psychological Outcome Measure	Results (baseline + end-of-intervention)	Key Findings
Bennett et al. (2024)	NCCN-Distress Thermometer (NCCN-DT; 0–10)	Baseline (categorical counts; score points, 0–10): n = 3 scored 5; n = 1 scored 4; n = 1 reported anxiety 7; n = 1 reported distress 1. End of intervention: not reported.	Baseline distress burden was common; NCCN-DT baseline scores ranged 1–5 with an anxiety rating up to 7, with no effect measured (no pre–post DT change was reported).
Bruns et al. (2018)	Overall quality of life (EORTC QLQ C29/30)	Baseline: median 58% (IQR 48–69). End of program: median 75% (IQR 65–83).	Quality of life showed moderate to large improvements; median QoL increased 58% → 75% (IQR 48–69 → 65–83, 17% increase), while depression risk was reported at baseline only (21%).
	Depression risk (GDS-2/15)	Baseline: n = 3/14 (21%) with GDS score >2 points (risk threshold). End of programme: not reported.	
Gkaintatzi et al. (2022)	HRQoL (EQ-5D VAS; 0–100)	Baseline: mean 69.53 VAS points (0–100). Post-rehabilitation: mean 85.71 VAS points.	HRQoL showed large improvements post-rehabilitation; mean EQ-5D VAS increased 69.53 → 85.71 (0–100, + 16.18 points) with a reported mean EQ-5D utility gain of +0.188 (0–1, 26% increase).
	EQ-5D utility index (0–1)	Baseline: not explicitly reported in extracted text. Post-rehabilitation: absolute value not explicitly reported; mean change reported as +0.188 utility index units (0–1; 26% increase).	
Li et al. (2024)	Hospital Anxiety Scale (HAS)	Baseline: mean ± SD HAS score (points): control 4.48 ± 2.37; intervention 4.18 ± 1.94 (between-group p = 0.503). End of intervention (day before surgery): mean ± SD HAS score (points): control 6.02 ± 2.37; intervention 2.94 ± 1.22 (p < 0.001). Control 4.90 ± 2.64; Intervention 2.04 ± 1.47 (p < 0.001)	Compared with control, prehabilitation was associated with large reductions in anxiety and moderate to large improvements in health status at end-of-intervention (HAS 2.94 ± 1.22 vs 6.02 ± 2.37; SF-12v2 84.37 ± 5.56 vs 74.70 ± 9.76), and early post-op recovery showed small to moderate improvements on QoR-9 by ~1.7–2.3 points on days 1–3.
	SF-12v2 total score	Baseline: mean ± SD SF-12v2 score (points): control 81.03 ± 9.29; intervention 81.30 ± 9.16 (between-group p = 0.882). End of intervention (day before surgery): mean ± SD SF-12v2 score (points): control 74.70 ± 9.76; intervention 84.37 ± 5.56 (p < 0.001). Control 63.88 ± 8.54; Intervention 77.92 ± 8.98 (p < 0.001)	
	QoR-9 (0–18; higher is better recovery)	Post-op days 1–3 (mean ± SD QoR-9 points): day 1 control 6.1 ± 2.2 vs intervention 7.8 ± 2.3; day 2 9.0 ± 2.7 vs 11.2 ± 2.7; day 3 11.3 ± 1.8 vs 13.6 ± 2.4 (all p < 0.001).	
MacDonald et al. (2020)	ESAS-r Depression (0–10)	Baseline (T1): mean (SE) ESAS-r depression score (points, 0–10) 2.08 (0.39). Post-intervention (T2): mean (SE) 1.90 (0.39); p = 0.478.	Mood symptoms showed minimal to no change post-intervention while disability showed small improvements: ESAS-r depression 2.08 → 1.90, anxiety 2.54 → 2.23, wellbeing 3.14 → 3.41 (0–10 points; all non-significant), and WHODAS 2.0 decreased 9.84 → 8.17 points (p = 0.03).
	ESAS-r Anxiety (0–10)	Baseline (T1): mean (SE) ESAS-r anxiety score (points, 0–10) 2.54 (0.47). Post-intervention (T2): mean (SE) 2.23 (0.42); p = 0.202.	
	ESAS-r Wellbeing (0–10)	Baseline (T1): mean (SE) ESAS-r wellbeing score (points, 0–10) 3.14 (0.39). Post-intervention (T2): mean (SE) 3.41 (0.46); p = 0.808.	
	WHODAS 2.0 disability	Baseline (T1): mean (SE) WHODAS 2.0 score (points) 9.84 (1.14). Post-intervention (T2): mean (SE) 8.17 (1.01); p = 0.03.	
Moorthy et al. (2023)	Emotional Distress Scale – Distress	Baseline: median (IQR) distress score (points) 3 (0–5). Post: median (IQR) 1 (0–2); p = 0.04.	Distress showed moderate improvements after the programme; median distress decreased 3 → 1 points (IQR 0–5 → 0–2; p = 0.04), with small to minimal changes in anxiety (3 → 2) and no change in depression (1 → 1).
	Emotional Distress Scale – Anxiety	Baseline: median (IQR) anxiety score (points) 3 (0–5). Post: median (IQR) 2 (0–3); p = 0.22.	
	Emotional Distress Scale – Depression	Baseline: median (IQR) depression score (points) 1 (0–3). Post: median (IQR) 1 (0–2); p = 0.41.	
Piché et al. (2023)	HADS Anxiety symptoms	Baseline (T1): mean (SD) HADS anxiety score (points) 7.80 (3.12), n = 25. End of intervention (T2): mean (SD) 6.92 (3.49), n = 25; p = 0.118. Follow-up (T3): Mean 7.04 (SD 3.98), n = 24	Psychological symptoms showed small, non-significant improvements pre-surgery: HADS anxiety 7.80 → 6.92, depression 4.84 → 4.28, and total 12.64 → 11.20 points, while stress (single-item Likert 1–5) showed moderate reductions (3.6 → 2.88).
	HADS Depression symptoms	Baseline (T1): mean (SD) HADS depression score (points) 4.84 (3.77), n = 25. End of intervention (T2): mean (SD) 4.28 (3.60), n = 25; p = 0.490. Follow-up (T3): Mean 5.17 (SD 3.70), n = 24	
	HADS Total score	Baseline (T1): mean (SD) HADS total score (points) 12.64 (6.20), n = 25. End of intervention (T2): mean (SD) 11.20 (6.46), n = 25; p = 0.109. Follow-up (T3): Mean 12.21 (SD 7.11), n = 24	
	Stress level (single-item Likert)	Baseline (T1): mean (SD) stress score (points; Likert 1–5) 3.6 (0.99), n = 25. End of intervention (T2): mean (SD) 2.88 (1.01), n = 25; p = 0.095.	
Waller et al. (2022)	HADS Anxiety	Baseline: mean (95% CI) HADS anxiety score (points) prehab 5.8 (3.7–10.1) vs control 6.6 (3.2–10.1). End of intervention: mean change (95% CI) in points prehab −0.5 (−2.0 to +0.9) vs control −1.2 (−2.1 to −0.2); p = 0.415.	Wearable-delivered prehabilitation showed no effect on mental health versus control; HADS anxiety changed −0.5 vs −1.2 points and depression −1.4 vs −0.8 points (p > 0.4), with similar baseline scores (anxiety 5.8 vs 6.6; depression 3.1 vs 3.7).
Waller et al. (2022)	HADS Depression	Baseline: mean (95% CI) HADS depression score (points) prehab 3.1 (1.4–4.8) vs control 3.7 (1.5–6.0). End of intervention: mean change (95% CI) in points prehab −1.4 (−2.4 to −0.3) vs control −0.8 (−2.2 to +0.5); p = 0.484.	
Wu et al. (2021)	EQ VAS (self-rated health)	Baseline: median (IQR) EQ VAS score (VAS points, 0–100) 75 (65–86). Post: median (IQR) 80 (70–90); p = 0.001.	Patient-reported health and fatigue showed small to moderate improvements; EQ VAS increased 75 → 80 (0–100; p = 0.001) and FACIT-Fatigue increased 44 → 47 (0–52; p < 0.001), while EQ-5D utility index showed no effect (0.796 → 0.796; p = 0.092) and the Anxiety/Depression dimension was reported directionally only.
	FACIT-Fatigue (0–52)	Baseline: median (IQR) FACIT-Fatigue score (points, 0–52) 44 (38–48). Post: median (IQR) 47 (43–50); p = 0.000 (reported as p < 0.001 in narrative).	
	EQ-5D utility index value	Baseline: median (IQR) EQ-5D utility index (index units, 0–1) 0.796 (0.691–0.857). Post: median (IQR) 0.796 (0.725–1.000); p = 0.092.	
	EQ-5D-3L Anxiety/Depression dimension	Categorical EQ-5D-3L dimension: baseline and post-intervention category counts not provided in extracted text. Narrative indicates more “No problems” overall after prehabilitation, but more “Extreme problems” for Anxiety/Depression post-intervention.	

**Abbreviations used:**

*Outcome measures:* EORTC QLQ C29/30, European Organisation for Research and Treatment of Cancer Quality of Life Questionnaire Colorectal 29/Core 30; EQ-5D, EuroQol 5-Dimension; ESAS-r, Edmonton Symptom Assessment System-revised; FACIT, Functional Assessment of Chronic Illness Therapy; GDS, Geriatric Depression Scale; HADS, Hospital Anxiety and Depression Scale; HAS, Hospital Anxiety Scale; NCCN-DT, National Comprehensive Cancer Network Distress Thermometer; QoR-9, Quality of Recovery-9; SF-12v2, Short Form-12 Health Survey version 2; WHODAS, World Health Organization Disability Assessment Schedule.

*Statistical terms:* CI, confidence interval; IQR, interquartile range; SD, standard deviation; SE, standard error.

*General:* HRQoL, health-related quality of life; QoL, quality of life; VAS, visual analogue scale.

**Table 4:** Physical outcomes and results reported in included papers.

Study Reference	Physical Activity/ Function Measure	Results (baseline + end-of-intervention)	Key Findings
Bennett et al. (2024)	Incremental Shuttle Walk Test (ISWT; metres)	Change from baseline to post-programme: median +150 m (range +110 to +190 m) among completers.	Functional capacity showed large improvements among completers; observed changes ranged from ISWT +110 to +190 m (median +150 m) and 1-MSTST +1 to +14 reps (median +8) over ~5 weeks.
	One-Minute Sit-to-Stand Test (1-MSTST; repetitions in 60 s)	Change from baseline to post-programme: median +8 reps (range +1 to +14 reps) among completers.	
Bruns et al. (2018)	Short Physical Performance Battery (SPPB; points)	Baseline: median 6 points (IQR 5–10). End of programme: median 9 points (IQR 6–10). Difference +25%.	Functional performance showed moderate improvements overall with minimal change in strength; observed pre–post ranges included SPPB 6 → 9 points, Fried 3 → 2 points, 4-m gait speed time 6.5 → 5.9 s, and handgrip 19 → 18 kg over a median 26-day programme.
	Fried frailty score (points)	Baseline: median 3 points (IQR 2–3). End of programme: median 2 points (IQR 1–4). Difference +20% (improvement).	
	4-meter gait speed (time; seconds)	Baseline: median 6.5 s (IQR 4.7–8.0). End of programme: median 5.9 s (IQR 4.6–7.6). Difference +6% (faster = better).	
	Hand grip strength (HGS; kilograms)	Baseline: median 19 kg (IQR 16–25). End of programme: median 18 kg (IQR 18–24). Difference –1% (declined).	
	Exercise adherence (days/week)	Programme dose: median duration 26 days (IQR 19–31); exercise performed mean 6 days/week (86%).	
Li et al. (2024)	6-Minute Walk Test (6MWT; meters)	Baseline (day of admission): mean ± SD 6MWT distance: control 409 ± 62 m; intervention 389 ± 64 m. End of intervention (day before surgery): change vs baseline: control −15.15 m; intervention +38.27 m. 30-day outcome: Intervention: -13.23 m relative to baseline; 71.4% (35/49) returned to baseline capacity Control: -81.64 m relative to baseline; 97.9% (47/48) showed decreased capacity.	Prehabilitation showed small to moderate improvements in pre-op walking capacity versus control; observed peri-intervention change ranged from −15.15 m (control) to +38.27 m (intervention) relative to baseline over ~7 days of intervention.
MacDonald et al. (2020)	6-Minute Walk Test (6MWT; metres)	Baseline (T1): mean (SE) 469.0 m (16.72). Post-intervention (T2): mean (SE) 510.9 m (13.74); p < 0.001. Follow-up (T3): Mean 498.3 m (SE 15.88), 95% CI 465.8-530.8, n = 28.	Post-intervention function and activity showed moderate improvements: 6MWT increased 469.0 → 510.9 m (+41.9 m) and grip strength increased 58.5 → 61.6 kg (+3.1 kg), with self-reported physical activity (Godin Total LSI) showing moderate to large improvements (18.4 → 28.4 points, + 54% increase).
	Grip strength (kilograms)	Baseline (T1): mean (SE) 58.5 kg (3.57). Post-intervention (T2): mean (SE) 61.6 kg (3.59); p = 0.003. Follow-up (T3): Mean 58.2 kg (SE 3.49), 95% CI 50.9-65.4, n = 28.	
	Godin-Shephard Leisure-Time Physical Activity Questionnaire (Total LSI; points)	Baseline (T1): mean (SE) 18.4 (2.22). Post-intervention (T2): mean (SE) 28.4 (3.37); p = 0.007. Follow-up (T3): Mean 26.0 (SE 3.14), 95% CI 19.6-32.5, n = 28.	
	Godin-Shephard Leisure-Time Physical Activity Questionnaire (Moderate-to-strenuous LSI; points)	Baseline (T1): mean (SE) 9.26 (1.74). Post-intervention (T2): mean (SE) 18.6 (3.02); p = 0.003. Follow-up (T3): Mean 17.4 (SE 2.91), 95% CI 11.4-23.4, n = 28.	
Moorthy et al. (2023)	30-second sit-to-stand (30s STS; repetitions)	Baseline: median 14.5 reps (IQR 10.5–15.5). Post: median 16 reps (IQR 16–22); p = 0.02.	Mixed effects were observed; 30s STS showed small improvements (14.5 → 16 reps) while heart rate recovery showed moderate, non-significant improvements (10.5 → 15.5 bpm), with no clear change in MET-min/week (346.5 → 407) or steps/day (5179 → 4550).
	Heart rate recovery (HRR; beats per minute)	Baseline: median 10.5 bpm (IQR 7.5–14). Post: median 15.5 bpm (IQR 11–20); p = 0.24.	
	Self-reported physical activity (MET-minutes/week)	Baseline: mean (SD) 346.5 (362). Post: mean (SD) 407 (400); p = 0.64.	
	Daily step count (steps/day)	Baseline: mean (SD) 5179 (3204). Post: mean (SD) 4550 (3061); p = 0.55.	
Piché et al. (2023)	2-Minute Step Test (steps)	Baseline (T1): n = 22, mean (SD) 98.91 (26.81) steps. End of intervention (T2): n = 13, mean (SD) 117.77 (14.35) steps; model-estimated difference vs T1 + 23.54 (95% CI 8.42–38.66); p = 0.005. Follow-up (T3): n = 18, mean 109.61 (SD 18.83).	Functional test performance showed small to moderate improvements; observed changes included 2-min step test +23.54 steps (95% CI 8.42–38.66), 30s STS + 1.62 reps (95% CI 0.44–2.79), and moderate-intensity activity showed large improvements (+150 min/week, 95% CI 120–180).
	30-second sit-to-stand (30s STS; repetitions)	Baseline (T1): n = 22, mean (SD) 9.82 (2.44) reps. End of intervention (T2): n = 13, mean (SD) 10.92 (1.80) reps; model-estimated difference vs T1 + 1.62 (95% CI 0.44–2.79); p = 0.011. Follow-up (T3): n = 19, mean 9.84 (SD 2.34).	
	Weekly moderate-intensity physical activity volume (minutes/week)	Baseline (T1): n = 15, mean (SD) 28.00 (108.44) min/week. End of intervention (T2): n = 15, mean (SD) 160.00 (58.55) min/week; model-estimated difference vs T1 + 150.00 (95% CI 120.00–180.00); p = 0.001. Follow-up (T3): n = 15, mean 50.00 (SD 113.39).	
Steffens et al. (2023)	Physical activity category (IPAQ-SF)	Baseline distribution (no intervention outcomes measured): high 33.3%, moderate 46.7%, low 20.0%.	No intervention effects measured; physical activity was described at baseline only (no intervention effect estimates); reported distribution ranged from 20.0% low to 33.3% high activity.
Waller et al. (2022)	6-Minute Walk Test (6MWT; meters)	Baseline: mean (95% CI) prehab 520.9 m (450.4–591.3) vs control 482.6 m (433.3–532.0). End of intervention (day before surgery): mean (95% CI) prehab 606.5 m (528.7–684.3) vs control 495.9 m (454.3–537.4). Mean change (pre-op minus baseline): + 85.6 m (95% CI 18.1–153.2) vs + 13.2 m (95% CI −6.8 to 33.2); p = 0.014.	Remote tri-modal prehabilitation showed large improvements in functional capacity; 6MWT increased 520.9 → 606.5 m (+85.6 m) in prehab vs 482.6 → 495.9 m (+13.2 m) in control, and during-program activity levels showed moderate to large increases in vigorous minutes (36.1 vs 17.5 min/day) with no clear difference in step-count (8919 vs 7961).
	Daily vigorous-intensity activity (minutes/day)	During prehabilitation period: mean (95% CI) prehab 36.1 min/day (21.2–50.9) vs control 17.5 min/day (5.2–29.7); p = 0.022.	
	Daily moderate-intensity activity (minutes/day)	During prehabilitation period: mean (95% CI) prehab 25.1 min/day (9.8–40.4) vs control 13.1 min/day (6.0–20.3); p = 0.063.	
	Daily step count (steps/day)	During prehabilitation period: mean (95% CI) prehab 8919 steps/day (7024–10814) vs control 7961 (5314–10608); p = 0.519.	
Wu et al. (2021)	FACIT-Fatigue (0–52 points)	Baseline: median (IQR) 44 (38–48). Post: median (IQR) 47 (43–50); p = 0.000.	Patient-reported fatigue and self-rated health showed small to moderate improvements; FACIT-Fatigue increased 44 → 47 points and EQ VAS increased 75 → 80 (0–100).
	EQ VAS (self-rated health; 0–100 VAS points)	Baseline: median (IQR) 75 (65–86). Post: median (IQR) 80 (70–90); p = 0.001.	

**Abbreviations used:**

Outcome measures: 1-MSTST, One-Minute Sit-to-Stand Test; 6MWT, 6-Minute Walk Test; EQ-5D, EuroQol 5-Dimension; EORTC QLQ C29/30, European Organisation for Research and Treatment of Cancer Quality of Life Questionnaire Colorectal 29/Core 30; FACIT, Functional Assessment of Chronic Illness Therapy; HGS, Hand Grip Strength; IPAQ-SF, International Physical Activity Questionnaire-Short Form; ISWT, Incremental Shuttle Walk Test; LSI, Leisure Score Index; SPPB, Short Physical Performance Battery; STS, Sit-to-Stand.

Statistical terms: CI, confidence interval; IQR, interquartile range; SD, standard deviation; SE, standard error.

General: bpm, beats per minute; MET, Metabolic Equivalent of Task; QoL, quality of life; QoR-9, Quality of Recovery-9; VAS, visual analogue scale.

**Table 5:** Other clinical outcomes reported in included papers.

Study Reference	Clinical Outcome/ QoL Measure	Results (baseline + intervention/perioperative)	Key Findings
Bruns et al. (2018)	Length of hospital stay (days)	Postoperative: 7 days (IQR 4–8).	QoL showed moderate to large improvements and perioperative outcomes were favourable; observed effects ranged from QoL + 17 percentage points (58% → 75%) with LOS 7 days (IQR 4–8), minor complications 21%, major 7%, and 30-day readmission 7%.
	Minor complications (Clavien–Dindo I–II)	Postoperative: 3/14 (21%).	
	Major complications (Clavien–Dindo III–IV)	Postoperative: 1/14 (7%).	
	30-day readmission	Postoperative: 1/14 (7%).	
Gkaintatzi et al. (2022)	Length of hospital stay (days)	Descriptive inpatient LOS for surgery pathway: mean 8.14 days (n = 97).	HRQoL showed large improvements in this service dataset, and mean hospital LOS was 8.14 days; however, outcomes are reported alongside ongoing cancer treatment and may not isolate prehabilitation effects.
Li et al. (2024)	Length of hospital stay (days)	Postoperative: mean (SD) control 6.4 (2.0) vs intervention 6.1 (2.6); p = 0.538.	Postoperative outcomes showed no effect between groups; LOS was 6.1 ± 2.6 vs 6.4 ± 2.0 days with identical complication grade counts (Grade 0–1: 46 vs 45; Grade 2: 3 vs 3; Grade 3 + : 0 vs 0).
	Postoperative complications (Clavien–Dindo grade counts)	Postoperative: Grade 0–1: control 45 vs intervention 46; Grade 2: 3 vs 3; Grade 3 + : 0 vs 0.	
	Early mobilization	Control: 1.7 ± 0.8 days; Intervention: 1.2 ± 0.6 days; p = 0.005.	
Moorthy et al. (2023)	Postoperative outcomes (complications, pneumonia, LOS)	Postoperative: all complications 13/26 (50%) vs 11/17 (64%), p = 0.30; postoperative pneumonia 6/26 (23%) vs 7/17 (41%), p = 0.12; hospital stay median (IQR) 10.5 (9–18) vs 17 (12.25–26) days, p = 0.07 (digital vs in-person).	Small, non-significant trends favoured the digital pathway, with observed ranges of pneumonia 23% → 41% and LOS 10.5 → 17 days, but differences were not statistically significant in this pilot.
Wu et al. (2021)	Length of hospital stay (days)	Post-treatment/surgery subgroup: median (IQR) LOS 3 (1–6) days among patients who underwent surgery (n = 53 with LOS available).	Descriptive outcomes only (no effect estimates); clinical outcomes were reported only descriptively for the surgical subset; LOS was 3 (1–6) days and 90-day readmission was 10/44 (23%) where data were available.
	90-day readmission	Post-treatment/surgery subgroup: 10/44 (23%) had a 90-day readmission (readmission data available for n = 44).	
	EQ VAS (self-rated health; 0–100 VAS points)	Baseline: median (IQR) 75 (65–86). Post: median (IQR) 80 (70–90); p = 0.001.	

**Abbreviations used:**

Outcome measures: EORTC QLQ C29/30, European Organisation for Research and Treatment of Cancer Quality of Life Questionnaire Colorectal 29/Core 30; EQ-5D, EuroQol 5-Dimension.

Statistical terms: CI, confidence interval; IQR, interquartile range; SD, standard deviation.

Clinical terms: Clavien-Dindo, surgical complication grading system (Grade I-V); LOS, length of stay; RCT, randomized controlled trial, EQ VAS, EuroQol Visual Analogue Scale; HRR, Heart Rate Recovery

General: HRQoL, health-related quality of life; QoL, quality of life; VAS, visual analogue scale.

In contrast to the varied psychological results, physical performance metrics consistently improved following multimodal digital prehabilitation regardless of delivery platform or cancer population. Walking capacity improvements represented the most consistently positive physical outcome across multiple interventions, with Six-Minute Walk Test performance improving significantly in mobile app interventions [37], hospital-based digital programmes [25], and wearable-based interventions [36]. Functional capacity, particularly as measured by Sit-to-Stand tests, improved across diverse digital delivery methods, including Zoom-based [34,35] and mobile app interventions [26]. Other objective measurements like grip strength showed statistically significant improvements [37], contrasting sharply with the non-significant changes in psychological parameters (depression, anxiety and well-being) within the same studies. Activity volume metrics showed differential responses based on intensity level. Moderate-to-vigorous physical activity increased significantly across three studies [35–37], while daily step counts showed less consistent improvements [26,36]. When comparing intervention and control groups, physical activity parameters demonstrated more consistent between-group differences than psychological measures. Digital prehabilitation consistently prevented decline in walking capacity that was observed in control participants [25,36]. The most substantial between-group differences in walking capacity were observed for a wearable-based intervention [36], suggesting wearable technology may particularly benefit objective physical performance.

#### Hospital stays and complications.

Four studies [25,26,33,39] assessed the length of postoperative hospital stay among surgical patients (see Table 5). Li et al. [25] reported that their intervention group had a slightly shorter mean postoperative hospital stay compared to their control group (6.1 ± 2.6 days vs. 6.4 ± 2.0 days). Although the difference was modest, it suggests a potential benefit of a digital prehabilitation tool in reducing hospital stay duration. Similarly, Bruns et al. [33] found an average length of stay of 7 days (IQR 4–8) following their digital prehabilitation intervention. Gkaintatzi et al. [39] observed an average hospital stay of 8.14 days among participants who underwent prehabilitation, indicating consistent findings across different settings. Notably, Moorthy et al. [34] reported a significant difference in length of hospital stay between their digital and in-person prehabilitation groups. The digital group had a median hospital stay of 10.5 days, whereas the in-person group had a longer median stay of 17 days. This suggests that digital prehabilitation may be more effective in reducing hospital stay length compared to traditional in-person methods. Additionally, Steffens et al. [24] highlighted patient expectations regarding hospital stay; 52% of participants anticipated a reduction in their length of stay by 4 days or more due to the prehabilitation programme.

The effect of prehabilitation on postoperative complications was also explored in multiple studies. Bruns et al. [33] reported that 21% of participants experienced minor complications classified as Clavien-Dindo grades I–II, and 7% had major complications (grades III–IV). Additionally, there was a 7% readmission rate within 30 days post-surgery. In Li et al.’s study [25], postoperative complications were comparable between intervention and control groups. Both groups had three patients experiencing grade 2 complications according to the Clavien-Dindo classification, with most patients in each group (45 in control, 46 in intervention) experiencing minimal or no complications (grades 0–1). Moorthy et al. [26] found that their digital multimodal prehabilitation group had a lower postoperative pneumonia rate of 23% compared to 41% in the in-person group. Furthermore, overall postoperative complications were lower in the digital group, with 50% of participants experiencing complications versus 64% in the in-person group. Steffens et al. [24] reported high patient expectations for improved postoperative outcomes, with 68% expecting at least a 50% reduction in complications due to the prehabilitation programme. While Waller et al. [36] suggested the potential for prehabilitation to reduce complications, they also noted that additional research is necessary to substantiate these findings.

### Integration of qualitative and quantitative results

Five studies [24,32,35,37,38] explored people’s views and experiences of digital multimodal prehabilitation interventions using qualitative methods. Of these, only Wu et al. [32] adopted a purely qualitative design, using the Braun and Clarke method for thematic analysis and deductive content analysis to map themes against the COM-B (Capability, Opportunity, Motivation, Behaviour) model of behaviour change with 22 semi-structured interviews. MacDonald et al. [37] conducted a mixed-methods pilot study with qualitative interviews (n = 9) to assess feasibility and acceptability using deductive and inductive coding. Steffens et al. [24] explored consumer perspectives from 30 gastrointestinal cancer patients using thematic framework analysis of open-ended survey responses. Piché et al. [35] assessed intervention fidelity through interviews with participating kinesiologists (n = 3) only. Bennett et al. [34] described qualitative content analysis but did not specify the analytical method used. Thematic synthesis of these qualitative findings revealed four overarching themes that explained patients’ experiences with digital multimodal prehabilitation: (1) accessibility and convenience of remote delivery, (2) technical and digital literacy challenges, (3) the critical role of human connection and support, and (4) personalisation and self-efficacy development. The analysis can be found in S1 Appendix. Following JBI convergent segregated methodology [40], quantitative and qualitative findings were integrated using a convergence matrix approach to identify areas of agreement, partial agreement, dissonance, and complementarity.

#### High satisfaction and acceptability (CONVERGENT).

Quantitative evidence demonstrated consistently high satisfaction across studies. All participants expressed satisfaction in Bennett et al.’s study [34], with 100% valuing exercise and diet interventions. Piché et al. [35] reported 100% overall satisfaction and willingness to recommend the programme. Waller et al. [36] found 100% rated their programme as “Good” or “Excellent.” Bruns et al. [33] reported a median satisfaction grade of 8/10, while Steffens et al. [24] achieved an app rating of 4.5/5, with 79% willing to recommend the programme. This was strongly corroborated by qualitative findings. MacDonald et al, [37] reported participants were “satisfied with the programme and its structure and glad they took part and found the team very supportive (p. 11).” In Wu et al.’s [32] study, participants described their overall experiences as positive, with the service meeting their needs and expectations. Participants across all five qualitative studies [24,32,35,37,38] expressed gratitude for programme participation and appreciation for the holistic support provided.

#### Accessibility and convenience benefits (CONVERGENT).

Quantitative evidence showed Waterland et al. [23] found 77% of their participants preferred online sessions over hospital-based education, with 97% finding the system easy to use. Bennett et al. [34] reported the majority of their sample found that the use of Zoom enhanced accessibility. MacDonald et al. [37] noted 89% of their participants logged into the app with minimal technical issues. This aligns closely with the findings of our qualitative synthesis, which revealed “accessibility and convenience of remote delivery” as a prominent cross-cutting theme. Wu et al. [32] identified that participants valued flexibility around schedules and avoiding hospital travel costs. MacDonald et al. [37] found their participants appreciated “being able to take part in the program from home rather than having to travel to the hospital" (p.11) and valued the convenience of home-based delivery.

#### Technical challenges and support needs (CONVERGENT).

Quantitative evidence also revealed variable technical challenges. Steffens et al. [24] found 21.4% needed technical support, despite 92% finding instructions easy. Moorthy et al.'s [26] participants reported experiencing initial connectivity issues with wearables, with data reliability improving from 25% to 86% due to iterative developments to the technology and user training. Some of Bruns et al.’s [33] participants reported touchscreen difficulties (14%) and technical issues (7%). Wu et al. [38] observed a 28% dropout over the course of their intervention, with feedback citing digital literacy issues. Adherence varied substantially across technology components. For example, MacDonald et al. [37] found drop-off in e-module completion despite 87% Fitbit usage among their participants. Qualitative findings strongly confirmed these patterns, with “technical and digital literacy challenges” identified as a significant barrier in the qualitative synthesis. Wu et al. [38] reported that participants mentioned lack of digital skills or confidence in using digital devices and noted cost barriers, with some lacking necessary devices or internet connectivity. Participants in Steffens et al.’s study [24] identified “poor preoperative health,” “lack of motivation,” and “lack of personal encouragement” as barriers, with some lacking confidence with mobile devices. MacDonald et al. [37] found participants had mixed feelings toward e-modules, finding them too long or lacking time to complete despite reminders.

#### Most valued intervention components (PARTIAL DISSONANCE → COMPLEMENTARITY).

Quantitative evidence suggested exercise and nutritional components were highly valued. Bennett et al. [34] found 100% of their participants valued exercise and diet interventions. Waller et al. [36] found 100% of their sample rated the exercise component as “Good” or “Excellent,” although dietary feedback was mixed (62% found it helpful) and the mindfulness app received varied feedback. Participants in MacDonald et al.'s study [37] reported improvements in strength, energy, and physical function. However, qualitative evidence revealed a more nuanced picture. For example, participants in MacDonald et al.'s study [37] found health coaching calls were “a valuable program component that encouraged accountability and provided an appreciated human touch element" (p.12), and were considered “the most valuable aspect” of the programme. Participants in Wu et al.'s study [32] identified the “patient-professional relationship” as a key motivator, with regular one-to-one interactions enabling trust and rapport building.

## Discussion

This systematic review aimed to assess the feasibility and effectiveness of digitally delivered multimodal prehabilitation interventions for cancer patients. Specifically, it sought to evaluate the feasibility and acceptability of such interventions; assess their impact on patient outcomes such as physical fitness, psychological readiness, nutritional status, and QoL; and explore patient and provider perspectives on their use in cancer care. The findings from the 12 included studies provide valuable insights into the potential of multimodal digital prehabilitation in oncology, highlighting both the promise and challenges associated with such interventions.

Significant heterogeneity in the design and implementation of interventions used across studies was evident. Variability in prehabilitation components such as exercise protocols, psychological support strategies, and nutritional guidance likely influenced the outcomes. For instance, some studies incorporated highly personalised, technology-driven interventions such as wearable devices and individualised counselling, while others relied on more generalised, non-individualised approaches. This lack of standardisation underscores the need for consensus on intervention design to ensure more reliable and comparable results in future studies. Importantly, the patient populations studied predominantly included those with colorectal, breast, and abdominal cancers, with limited representation of individuals with liver and hepatobiliary cancers, lung cancer, and brain or head and neck cancers. These latter groups may have unique needs due to the higher levels of functional and psychological impairment often associated with their conditions [41,42]. Given the increased burden of symptoms and functional limitations, there is an urgent need for the development and evaluation of digitally delivered multimodal prehabilitation tailored specifically to these populations.

Within the current review, digitally delivered multimodal prehabilitation programmes consistently demonstrated efficacy in improving physical fitness among cancer survivors. Robust gains were observed in walking capacity, functional strength, and overall physical activity across multiple studies. Interventions that incorporated personalised exercise regimens tailored to individual baseline fitness or functional assessment and delivered via platforms such as Zoom, social media platforms like WeChat, or dedicated mobile applications were particularly effective, yielding significant improvements in standardised measures such as the Six Minute Walk Test, Incremental Shuttle Walk Test, and Sit to Stand tests. For instance, Li et al. [25] reported a 38.27-metre improvement in Six Minute Walk Test distance, while MacDonald et al. [37] observed a 41.96-metre gain at eight weeks, both exceeding typical postoperative declines in control groups. Likewise, enhanced engagement and adherence, facilitated by real-time feedback through apps and wearables, further contributed to these positive outcomes, as seen in studies utilising wearable devices for activity monitoring and feedback [26,36]. Importantly, the impact of personalisation emerged as a critical determinant of exercise effectiveness, with studies [25,26,38] that adopted individualised exercise prescriptions consistently reporting better physical outcomes than studies that adopted generic or semi-personalised protocols. By acknowledging the heterogeneity in baseline fitness, comorbidities, and functional capacity among cancer patients, individualised multimodal digital prehabilitation approaches enable tailored exercise dosing that maximises physiological adaptation while minimising injury risk in oncological populations with varying degrees of deconditioning [43].

A notable pattern emerging from this review is a consistent improvement in physical outcomes across the included studies, contrasted with more variable and in some cases negative changes in psychological outcomes. Although prior research underscores the importance of digital psychological support in cancer care [8,44], these mixed findings suggest that psychological components within multimodal digital prehabilitation require careful design. A comparison of studies based on psychological intervention design suggests potential differences in effectiveness. Only four of the 12 included studies implemented individualised psychological interventions such as tailored counselling, motivational messaging, or relaxation techniques adapted to patient needs [34,35,38,39], and reported more favourable changes in anxiety and distress than those using generic or pre-recorded content. For example, Li et al. [25] delivered personalised psychological support via WeChat, incorporating breathing exercises, muscle relaxation, meditation, and motivational messages, and reported significant anxiety reduction (Hospital Anxiety Scale: 4.18 ± 1.94 to 2.94 ± 1.22, p < 0.001), whereas anxiety worsened in controls. Similarly, studies incorporating live group counselling or interactive coaching calls tended to report improved psychological outcomes and higher engagement among participants [34,37–39], although these findings should be interpreted with caution given the small sample sizes and variability in study quality involved. In contrast, generic psychological interventions appeared to produce more variable effects, with several studies reporting minimal or non-significant changes. For example, Waller et al. [36] used a pre-recorded mindfulness app and observed no significant changes in anxiety or depression, with participants reporting low perceived usefulness. Likewise, Piché et al. [35] reported non-significant improvements in anxiety and depression following generic educational psychological support. These findings align with the broader literature on digital mental health, which indicates that personalisation through tailored feedback, adaptive content, and self-monitoring substantially enhances effectiveness [45,46].

Another key finding of this review was that intervention intensity, duration, theoretical grounding, and digital modality emerged as key determinants of psychological outcomes. Higher-intensity interventions featuring live, interactive engagement with healthcare professionals generally showed more favourable outcomes than lower-intensity, self-directed approaches, based on within-study comparisons. For example, Bennett et al. [34] delivered twice-weekly, hour-long group counselling sessions via Zoom over five weeks, while MacDonald et al. [37] provided weekly individualised coaching calls across eight weeks, with participants in both studies reporting high levels of satisfaction and engagement. Meta-analytic evidence from the broader digital health literature suggests that interventions lasting 12 weeks or longer may be most effective for improving anxiety, depression, and fatigue in cancer populations [47]. Most of the studies included in the current review had interventions of shorter durations, however, which may reduce acute anxiety but typically show limited effects on depression [48]. A notable gap identified in this review is the inconsistent reporting of the theoretical frameworks underpinning the interventions delivered. Only three studies [34,37,38] explicitly articulated a guiding theory, although Cognitive Behavioural Therapy (CBT), mindfulness, and motivational approaches were implicitly reflected in several of the other interventions. This mirrors wider trends in digital psychosocial oncology, where over half of interventions described in the literature lack an explicit theoretical basis [49]. Although digital CBT interventions have shown promise for reducing anxiety and depression in cancer patients and mindfulness-based approaches may help to reduce fear of cancer recurrence [49–51], these specific approaches were inconsistently implemented in the included prehabilitation studies. The diversity of digital modalities employed, including live videoconferencing, mobile applications, wearables, and online resources, is likely to have contributed to outcome variability, although none of the included studies directly compared modality-specific effects.

Beyond symptom reduction, digital multimodal prehabilitation may offer broader mental health benefits. Several studies reported meaningful improvements in psychological wellbeing, while qualitative findings indicated that some participants experienced reduced isolation, increased confidence, enhanced perceived control, and improved psychological preparedness for surgery as a result of their interventions [24,32,34,37]. These outcomes are particularly relevant given that 30–40% of cancer patients experience clinically significant levels of anxiety or depression, with a higher prevalence being observed in the preoperative period [52,53]. The multimodal nature of these interventions, especially the exercise component, may have confer additive psychological benefits through neurobiological mechanisms such as improved sleep and reduced inflammation [54,55], and psychosocial factors such as enhanced self-efficacy and therapeutic alliance [56,57]. Importantly, adherence rates were high across studies, contrasting with the substantial attrition often reported in digital-only psychological interventions [58]. Given established links between preoperative psychological distress and adverse postoperative outcomes, delayed recovery, increased pain, prolonged stays, and higher complications [59,60], interventions that successfully reduce anxiety and build psychological resilience among cancer patients could potentially offer downstream benefits for recovery and survivorship, although this remains to be definitively demonstrated in the prehabilitation context. This potential may be best realised through interventions that prioritise personalisation, theoretical rigour, and integrated design, as generic psychological components generally showed more limited effectiveness in the included studies.

Nutritional status was critically underreported across studies, representing a substantial evidence gap in current digital multimodal prehabilitation research. Among the 12 included studies, only one [33] systematically assessed nutritional status using the Mini Nutritional Assessment (MNA), revealing that 64% (9/14) of frail elderly patients were at risk for malnutrition at baseline. While nutritional education and personalised diet plans were common intervention components, the impact on objective nutritional outcomes such as lean body mass, body composition, or validated nutritional indices was rarely measured or reported. Only Waller et al. [36] reported participants’ body weight changes, which remained minimal (mean change: + 0.46 kg intervention vs −1.06 kg control), although body weight alone is a crude indicator that fails to distinguish between lean mass preservation and fat mass changes [36].

This represents a critical missed opportunity, particularly given compelling evidence from meta-analyses demonstrating that skeletal muscle loss during cancer treatment is associated with a 3.13-fold higher mortality risk [61], increased treatment toxicity [61,62], and functional impairment [63]. Combined exercise and nutritional interventions have demonstrated efficacy in preserving lean mass and improving body composition in cancer patients [63,64], with multimodal prehabilitation specifically reducing skeletal muscle loss during neoadjuvant therapy in oesophageal cancer patients [65]. The magnitude of this gap is underscored by systematic reviews of prehabilitation research, which consistently identify inconsistent nutritional assessment, reliance on non-validated tools, and minimal reporting of body composition changes as pervasive methodological limitations [66,67]. Established cancer nutrition guidelines, including European Society for Clinical Nutrition and Metabolism (ESPEN) recommendations for protein intake (1.0-1.5 g/kg/day) [68], ESPEN guidelines on nutritional support within Enhanced Recovery After Surgery protocols [69], and consensus statements on sarcopenia prevention [70], provide clear frameworks for standardised assessment, yet these were not applied in the included studies. Future research should prioritise rigorous nutritional outcome measurement using validated tools such as the Patient-Generated Subjective Global Assessment (PG-SGA), paired with objective body composition assessment like dual-energy X-ray absorptiometry (DXA), computed tomography (CT), or bioimpedance analysis to quantify changes in skeletal muscle mass, lean body mass, and fat mass [66,67]. Without such standardisation, the true effectiveness of the nutritional component within multimodal digital prehabilitation remains impossible to ascertain, limiting evidence-based optimisation of these interventions and hindering translation into clinical practice.

Outcomes of several studies in this review suggest that digitally delivered multimodal prehabilitation may reduce hospital stay durations and postoperative complications. Especially among the comparative studies, individuals who participated in digital prehabilitation had shorter median hospital stays and fewer complications compared to those in the in-person group. The reduction in postoperative complications, especially pneumonia, suggests that multimodal digital prehabilitation interventions can effectively prepare patients for surgery by improving physical function and possibly immune resilience, thereby lowering hospital resource utilisation and improving postoperative recovery. These findings align with previous research indicating that prehabilitation can enhance postoperative recovery [71]. However, the limited number of studies reporting on these outcomes and the variability in methodologies employed highlight the need for more rigorous research to confirm these benefits.

Participant feedback generally indicated high levels of satisfaction with the interventions included in this review. Participants appreciated the convenience and flexibility of digital delivery, the ability to involve family members, and the comprehensive support provided. These positive perceptions are consistent with broader trends in digitally delivered patient-centred care and the growing acceptance of telehealth services [72,73]. The technological feasibility and usability of digitally delivered multimodal prehabilitation interventions were also generally reported as positive across the included studies. Some participants often found the digital platforms user-friendly and accessible, which may have facilitated their engagement with the interventions from their own homes. This ease of use likely contributed to the high adherence rates observed in several studies, as participants could integrate the programmes into their daily routines with minimal disruption.

However, some participants reported challenges with their multimodal digital prehabilitation programmes, such as scheduling flexibility, technological difficulties, and mixed feedback on certain components like mindfulness apps. For technical challenges, issues such as unclear user interfaces, device connectivity problems, touchscreen difficulties, a lack of confidence with mobile devices, and a significant need for technical support to navigate platforms effectively were reported across multiple studies. This suggests that while multimodal digital prehabilitation is feasible for many cancer patients, there is a subset who may struggle due to these technological barriers. Many factors may be implicated; for instance, a study by Zhang et al. [74] found that factors such as age, education level, and socioeconomic status affect e-health literacy among cancer survivors, with older patients and those with lower education levels often exhibiting reduced digital proficiency. Similarly, a review by López et al. [75] highlighted that disparities in digital literacy can hinder the effective implementation of digital health technologies in oncology care. Implementing strategies to enhance digital health literacy, such as providing technical support [76], simplifying user interfaces [77] or offering introductory sessions on technology use [72,73], not only improves individual patient experiences but also contributes to the overall success and scalability of digital health interventions in oncology.

Recruitment rates across the included studies varied widely, reflecting the interplay between programme design, user experience and participant accessibility. Studies that reported high user satisfaction and positive feedback [24,36,38] achieved notably high recruitment rates, with some reaching 100%. These interventions often featured user-friendly digital platforms, personalised support and strong participant engagement, all of which likely contributed to their success in attracting and retaining participants. Conversely, studies encountering technological barriers or challenges with digital literacy [32,33] tended to report lower recruitment rates or higher dropout, underscoring the importance of addressing usability and accessibility in digital health interventions. Socioeconomic factors also played a role, as highlighted by Piché et al. [35], who noted that limited internet access and scheduling conflicts impacted recruitment despite high satisfaction among those enrolled. Collectively, these findings emphasise that maximising recruitment in multimodal digital prehabilitation programmes for cancer patients requires not only effective intervention content but also careful attention to technological support, digital inclusivity, and the overall user experience.

Digital interventions have been shown to enhance patient engagement through personalised content and flexible access [58,78]. In the current review, dropout rates varied across studies, with some reporting no dropouts and others experiencing higher rates. These were influenced by factors such as treatment-related fatigue, technological challenges or a lack of engagement with the intervention components.

Notably, adherence rates were systematically higher in studies implementing personalised interventions compared to those using generic approaches. Studies incorporating tailored exercise prescriptions, individualised psychological support, and personalised nutritional guidance consistently reported adherence rates exceeding 85% [25,26,34,37]. Conversely, interventions employing more generic or semi-personalised components demonstrated more variable adherence patterns [33,35,36,38]. For instance, Bruns et al. [33], using generic dietary plans and social reward systems, reported 86% exercise adherence but only 71% dietary adherence. It is also interesting to note that MacDonald et al. [37] observed substantial drop-off in generic e-module completion among participants despite high engagement with personalised coaching calls. This pattern aligns with broader eHealth literature demonstrating that tailored digital interventions achieve 1.5–2 times higher engagement and adherence than non-tailored approaches [79,80]. These findings suggest that personalisation is not merely an enhancement but a fundamental determinant of sustained patient engagement in digitally delivered multimodal prehabilitation programmes.

However, challenges in adherence were noted in specific components of some of the included interventions, particularly the psychological component. For example, Waller et al. [36] reported that participants struggled to perceive the relevance of the psychological component of their intervention, with some finding the mindfulness app provided unhelpful. This suggests that user experience and the perceived relevance of programme components may significantly impact adherence to digital interventions. Similarly, Linardon et al. [58] highlighted that adherence to digital psychological interventions is a significant challenge, where a 24% attrition rate was reported and 34% were lost to follow-up in their study. Therefore, ensuring all components of multimodal digital prehabilitation programmes are engaging and perceived as beneficial by participants is crucial for improving adherence and maximising the effectiveness of these interventions. One way to achieve this is by incorporating co-design approaches in intervention development, which can significantly enhance the relevance, engagement, and user-friendliness of multimodal digital prehabilitation programmes for cancer patients [81]. While co-design approaches hold considerable promise for enhancing intervention relevance and engagement, our review found that only a quarter of the included studies explicitly incorporated patient or stakeholder involvement in intervention development. Moorthy et al. [26] involved a patient user group and clinical team in co-designing remote assessment tools, Bennett et al. [34] engaged a patient and public involvement (PPI) representative in their organising committee, and Bruns et al. [33] conducted patient interviews to inform programme adaptations. This represents a significant missed opportunity, as human-centred participatory design involving patients, caregivers, and healthcare professionals as equal partners throughout iterative development cycles has been shown to enhance intervention usability, relevance and adoption, symptom management, and potentially overall survival through timely side-effect management, as well as improving patient satisfaction and long-term sustainability [82–84].

Established co-design frameworks provide practical roadmaps for future digitally delivered prehabilitation research. For example, the UK Standards for Public Involvement and the EQUATOR Network's Guidance for Reporting Involvement of Patients and the Public (GRIPP), now in its second iteration, provide structured methodologies for meaningful patient engagement, emphasizing early involvement, shared decision-making, and sustained partnership rather than token consultation [84,85]. In telehealth and digital prehabilitation contexts, participatory co-design involves multi-stakeholder workshops, user experience testing, iterative prototyping, and continuous feedback loops to ensure interventions address locally relevant problems while remaining inclusive across diverse patient populations, digital literacy levels, and access to resources [2,86]. Future digital multimodal prehabilitation trials should integrate co-design from inception, especially in designing intervention components. Without such meaningful engagement, digital interventions risk failing to reach those with the greatest need, lacking contextual relevance, or suffering from poor adherence due to a mismatch between intervention design and patient realities [83,87].

The digital divide represents a critical equity challenge requiring proactive attention in future digital prehabilitation research and implementation. Evidence from included studies suggests that socioeconomic status, geographic location, and language create systematic, intersecting barriers that can fundamentally limit access to digitally delivered interventions. Socioeconomic disparities operate through multiple pathways. For example, 32% of participants in one study reported household incomes below $50,000 with internet access explicitly identified as an accessibility barrier [36], while in another cohort, 28.5% earned less than $20,800 annually, with 40% lacking tablet/computer ownership and 5% without smartphone [31]. Device ownership gaps required Waller et al. [36] to implement lending schemes after finding 27% of participants lacked compatible devices. However, device access represents only one dimension, with ongoing costs of data plans and broadband subscriptions imposing sustained financial burdens disproportionately affecting lower-income patients already facing treatment-related financial strain [88,89]. Digital literacy, which is strongly correlated with both socioeconomic status and education, compounds these barriers. For example, in one study, 20% of those declining digital services cited lack of technology confidence while 30% identified language problems [26]. Geographic location emerged as a second systematic barrier, with 69% of participants in one study residing in rural/regional areas and 77% preferring telehealth to avoid travel burdens [23]. Yet rural communities experience significantly lower broadband access globally, with 22.3% of rural Americans lacking high-speed internet (25/3 Mbps) compared to only 1.5% in urban areas, while the European Union shows a narrower but persistent gap with 9.5% of rural households lacking internet access compared to 5.1% in urban areas [90,91]. The above is creating a paradox where those benefiting most from digitally delivered interventions face the greatest connectivity barriers. Addressing these inequities demands equity-focused research designs grounded in established frameworks, such as the Framework for Integrating Telehealth Equitably (FITE), which emphasizes multilevel determinants beyond access alone [92], or the Digital Health Equity Framework (DHEF), which provides structured guidance for embedding equity throughout intervention lifecycles, addressing digital determinants at individual, interpersonal, community, and societal levels [88,89]. In order to address such inequities, future trials should actively prioritise underrepresented group enrolment through targeted outreach, while eliminating exclusionary eligibility criteria [88,93], incorporate qualitative inquiry with underserved participants [94], and integrate equity-enhancing supports as core components rather than optional add-ons [88,95].

### Strengths and limitations

To the authors’ knowledge, this is the first systematic review on the feasibility, acceptability and effectiveness of multimodal digital prehabilitation in cancer survivors. A multidisciplinary team, including those with backgrounds in psychology and physiotherapy, carried out the review. We used JBI critical appraisal tools to comprehensively evaluate the studies, allowing for in-depth analysis of various research designs, and applied the TIDieR checklist to systematically document intervention characteristics and identify reporting deficiencies. This structured approach enhanced the clarity of our findings and underscored critical areas for future research. Nonetheless, this systematic review has several limitations that warrant consideration. First, the field of digitally delivered multimodal prehabilitation is in its nascent stages, with numerous ongoing protocols and pilot studies. Our findings therefore represent a snapshot of a rapidly growing evidence base, with further systematic reviews needed to capture emerging studies and provide updated insights. Second, study quality and design limitations affected the certainty of our findings. Most included studies (n = 10/12) employed single-arm designs without control groups, limiting our ability to attribute observed improvements to the interventions rather than other factors such as placebo effects or natural recovery. Only one randomized controlled trial was included [36], which lacked outcome assessor blinding. Additionally, the majority of studies were small feasibility trials (with sample sizes of 6–57 participants in most cases), and thus susceptible to “small study effects” that may introduce bias toward positive findings. Due to the limited number of studies, we were unable to construct funnel plots to formally assess publication bias. We addressed these limitations by conducting a narrative synthesis rather than meta-analysis, prioritising patterns across multiple independent studies, and refraining from strong causal claims. We also gave greater weight to findings from higher-quality studies and explicitly noted when conclusions relied on single or lower-quality studies.

Third, several methodological issues limit the generalisability of the included studies’, and consequently this review’s, findings. Poor recruitment rates in some studies (29–49%) suggest potential selection bias, with enrolled participants likely being more digitally literate or motivated than those who declined. Single-centre recruitment in most studies further restricts the generalisability of their findings. One study reported substantial data collection issues and mid-study intervention modifications [26], which we considered when interpreting their findings. Despite these limitations, the high adherence rates (70–100%) consistently observed across multiple independent studies strengthens confidence that digital prehabilitation is feasible for appropriately selected cancer patients. Fourth, significant heterogeneity in intervention designs, outcome measures, and assessment tools precluded quantitative synthesis. Variability in exercise protocols, psychological support strategies, and nutritional guidance limits comparability across studies and underscores the need for standardisation in future research. Finally, participants in the included studies were predominantly those with colorectal, breast, and abdominal cancers, with limited representation of individuals with liver and hepatobiliary cancers, lung cancer, and head and neck cancer. Applying the findings of this review to these under-represented patient groups may therefore not be appropriate, with further research needed to demonstrate the efficacy of digital prehabilitation in this cohort.

### Implications for cancer care research, practice and policy

The findings of this systematic review have significant implications for cancer care research, clinical practice and health policy. For researchers, the considerable heterogeneity observed in intervention designs highlights the urgent need for standardisation in digitally delivered multimodal prehabilitation studies. Establishing consensus on core components based on cancer types, such as exercise protocols, psychological support, and nutritional guidance, and standardising outcome measures will enhance comparability across studies and strengthen the evidence base. Additionally, the inconsistent reporting of nutritional outcomes and mixed psychological results indicate critical gaps that future research should address. Emphasising co-design approaches that involve patients, carers and healthcare professionals in the development of interventions can improve relevance and engagement, leading to better adherence and outcomes [87]. Researchers should also prioritise the inclusion of under-represented populations, such as different cancer types, to ensure that findings are generalisable and that interventions are tailored to the unique needs of all cancer patients.

From a clinical practice and policy perspective, positive patient feedback and potential reductions in hospital stays and postoperative complications suggest that digitally delivered multimodal prehabilitation could be a valuable addition to standard cancer care. Clinicians should consider integrating these interventions into treatment pathways, with a focus on personalisation and patient engagement, to enhance effectiveness. However, to maximise the benefits and ensure equitable access, policymakers must address barriers related to digital literacy and socioeconomic disparities. This includes investing in digital infrastructure, providing technical support, and implementing educational programmes to improve e-health literacy among patients. Policies should also promote the development of guidelines and best practices for the design and implementation of digital health interventions in oncology. By aligning research efforts, clinical practice, and health policy, stakeholders can work collaboratively to optimise the delivery of digital prehabilitation, ultimately improving patient outcomes and advancing the quality of cancer care.

## Conclusion

Digitally delivered multimodal prehabilitation interventions hold significant promise for transforming cancer care by enhancing patients’ physical fitness and preparedness for treatment, as demonstrated in this systematic review. Despite challenges such as heterogeneity in intervention designs and inconsistent reporting, particularly regarding psychological and nutritional outcomes, the high levels of patient satisfaction and engagement affirm the feasibility and acceptability of these digital programmes. The identified barriers, including technological difficulties and varying levels of digital literacy, underscore the imperative for standardised, co-designed interventions that are tailored to individual patient needs and capabilities. By addressing these challenges and integrating personalised digital prehabilitation into standard oncology practice, there is a compelling opportunity to not only improve patient outcomes and QoL but also to reduce hospital stays and postoperative complications. This underscores the need for concerted efforts in research, clinical implementation, and policy development to harness the full potential of digital prehabilitation as a catalyst for advancing patient-centred cancer care.

## Supporting information

S1 Checklist PRISMA checklist. From: Page MJ, McKenzie JE, Bossuyt PM, Boutron I, Hoffmann TC, Mulrow CD, et al. The PRISMA 2020 statement: an updated guideline for reporting systematic reviews. BMJ 2021;372:n71. https://doi.org/10.1136/bmj.n71. This work is licensed under CC BY 4.0. To view a copy of this license, visit https://creativecommons.org/licenses/by/4.0/.
(DOCX)

S1 Appendix Search queries, quality appraisal and qualitative findings. (DOCX)

S2 Appendix Title and abstract screening for all included studies. (XLSX)

S3 Appendix Codebooks, TIDieR and data extraction for all included studies. (XLSX)

## References

[1] Stout NL, Fu JB, Silver JK. Prehabilitation is the gateway to better functional outcomes for individuals with cancer. J Cancer Rehabil. 2021;4:283–6. 35048084 PMC8765744

[2] Wernick R, Issokson K, Banty A, Castelan VC, Gwarnicki C, Solomon T, et al. Multimodal prehabilitation may improve surgical outcomes for patients with inflammatory bowel disease. Inflamm Bowel Dis. 2024;30(Supplement_1):S6–7. doi: 10.1093/ibd/izae020.014

[3] Guerra-Londono CE, Cata JP, Nowak K, Gottumukkala V. Prehabilitation in adults undergoing cancer surgery: a comprehensive review on rationale, methodology, and measures of effectiveness. Curr Oncol. 2024;31(4):2185–200. doi: 10.3390/curroncol31040162 38668065 PMC11049527

[4] Scheede-Bergdahl C, Minnella EM, Carli F. Multi-modal prehabilitation: addressing the why, when, what, how, who and where next? Anaesthesia. 2019;74 Suppl 1:20–6. doi: 10.1111/anae.14505 30604416

[5] Bingham SL, Small S, Semple CJ. A qualitative evaluation of a multi-modal cancer prehabilitation programme for colorectal, head and neck and lung cancers patients. PLoS One. 2023;18(10):e0277589. doi: 10.1371/journal.pone.0277589 37788238 PMC10547201

[6] Fabi A, Rossi A, Mocini E, Cardinali L, Bonavolontà V, Cenci C, et al. An integrated care approach to improve well-being in breast cancer patients. Curr Oncol Rep. 2024;26(4):346–58. doi: 10.1007/s11912-024-01500-1 38400984 PMC11021235

[7] Rossen S, Kayser L, Vibe-Petersen J, Christensen JF, Ried-Larsen M. Cancer survivors’ receptiveness to digital technology-supported physical rehabilitation and the implications for design: qualitative study. J Med Internet Res. 2020;22(8):e15335. doi: 10.2196/15335 32755892 PMC7439140

[8] Harris J, Cheevers K, Armes J. The emerging role of digital health in monitoring and supporting people living with cancer and the consequences of its treatments. Curr Opin Support Palliat Care. 2018;12(3):268–75. doi: 10.1097/SPC.0000000000000362 29927756

[9] Signorelli GR, Lehocki F, Mora Fernández M, O’Neill G, O’Connor D, Brennan L, et al. A research roadmap: connected health as an enabler of cancer patient support. J Med Internet Res. 2019;21(10):e14360. doi: 10.2196/14360 31663861 PMC6914240

[10] Fuller TE, Pong DD, Piniella N, Pardo M, Bessa N, Yoon C, et al. Interactive digital health tools to engage patients and caregivers in discharge preparation: implementation study. J Med Internet Res. 2020;22(4):e15573. doi: 10.2196/15573 32343248 PMC7218608

[11] Kehagia AA, Chowienczyk S, Helena van Velthoven M, King E, North T, Shenton D, et al. Real-world evaluation of the feasibility, acceptability and safety of a remote, self-management Parkinson’s disease care pathway: a healthcare improvement initiative. J Parkinsons Dis. 2024;14(1):197–208. doi: 10.3233/JPD-230205 38250784 PMC10836560

[12] De Oliveira Trigo A, De Oliveira Trigo B, Khan Sullivan F. P-556 Digital platform for remote pre-treatment assessment: perceived usefulness and quality by patients and doctors. Hum Reprod. 2023;38.

[13] Cano I, Barberan-Garcia A, Iriso S, Solans O, Miralles F, Roca J, et al. Implementation of digital health tools for scalability of a prehabilitation service. Int J Integr Care. 2019;19(4):195. doi: 10.5334/ijic.s3195

[14] Fulop A, Lakatos L, Susztak N, Szijarto A, Banky B. The effect of trimodal prehabilitation on the physical and psychological health of patients undergoing colorectal surgery: a randomised clinical trial. Anaesthesia. 2021;76(1):82–90. doi: 10.1111/anae.15215 32761611

[15] Asada HH, Reisner A. Wearable sensors for human health monitoring. In: Tomizuka M, Yun C-B, Giurgiutiu V, editors. Proc SPIE 6174, Smart Structures and Materials 2006: Sensors and Smart Structures Technologies for Civil, Mechanical, and Aerospace Systems; 2006. 617401 p. doi: 10.1117/12.667764

[16] Casperson SL, Sieling J, Moon J, Johnson L, Roemmich JN, Whigham L. A mobile phone food record app to digitally capture dietary intake for adolescents in a free-living environment: usability study. JMIR Mhealth Uhealth. 2015;3(1):e30. doi: 10.2196/mhealth.3324 25775506 PMC4381810

[17] Agnisarman S, Narasimha S, Chalil Madathil K, Welch B, Brinda F, Ashok A, et al. Toward a more usable home-based video telemedicine system: a heuristic evaluation of the clinician user interfaces of home-based video telemedicine systems. JMIR Hum Factors. 2017;4(2):e11. doi: 10.2196/humanfactors.7293 28438724 PMC5422657

[18] Yang L, Wen-bo W, Xiong Y, et al. Mobile-based multimodal rehabilitation including exercise, nutritional, and psychological interventions in patients with abdominal cancer receiving concurrent chemoradiotherapy: prospective, multicenter, randomized trial. J Clin Oncol. 2023;41:e24138.

[19] Barberan-Garcia A, Cano I, Bongers BC, Seyfried S, Ganslandt T, Herrle F, et al. Digital support to multimodal community-based prehabilitation: looking for optimization of health value generation. Front Oncol. 2021;11:662013. doi: 10.3389/fonc.2021.662013 34249698 PMC8270684

[20] Piraux E, Caty G, Reychler G, Forget P, Deswysen Y. Feasibility and preliminary effectiveness of a tele-prehabilitation program in esophagogastric cancer patients. J Clin Med. 2020;9(7):2176. doi: 10.3390/jcm9072176 32660126 PMC7408844

[21] Gudmundsson GH, Mészáros J, Björnsdóttir ÁE, Ámundadóttir ML, Thorvardardottir GE, Magnusdottir E, et al. Evaluating the feasibility of a digital therapeutic program for patients with cancer during active treatment: pre-post interventional study. JMIR Form Res. 2022;6(10):e39764. doi: 10.2196/39764 36227639 PMC9614627

[22] Li C, Carli F, Lee L, Charlebois P, Stein B, Liberman AS, et al. Impact of a trimodal prehabilitation program on functional recovery after colorectal cancer surgery: a pilot study. Surg Endosc. 2013;27(4):1072–82. doi: 10.1007/s00464-012-2560-5 23052535

[23] Waterland JL, Chahal R, Ismail H, Sinton C, Riedel B, Francis JJ, et al. Implementing a telehealth prehabilitation education session for patients preparing for major cancer surgery. BMC Health Serv Res. 2021;21(1):443. doi: 10.1186/s12913-021-06437-w 33971869 PMC8108411

[24] Steffens D, Denehy L, Solomon M, Koh C, Ansari N, McBride K, et al. Consumer perspectives on the adoption of a prehabilitation multimodal online program for patients undergoing cancer surgery. Cancers (Basel). 2023;15(20):5039. doi: 10.3390/cancers15205039 37894406 PMC10605909

[25] Li X, Sha L, He Y, Yi J, Wang X. The impact of short-term multimodal prehabilitation on functional capacity in patients with gynecologic malignancies during the perioperative period: a prospective study. Eur J Oncol Nurs. 2024;70:102577. doi: 10.1016/j.ejon.2024.102577 38636115

[26] Moorthy K, Halliday LJ, Noor N, Peters CJ, Wynter-Blyth V, Urch CE. Feasibility of implementation and the impact of a digital prehabilitation service in patients undergoing treatment for oesophago-gastric cancer. Curr Oncol. 2023;30(2):1673–82. doi: 10.3390/curroncol30020128 36826089 PMC9955831

[27] Santos WMD, Secoli SR, Püschel VA de A. The Joanna Briggs Institute approach for systematic reviews. Rev Lat Am Enfermagem. 2018;26:e3074. doi: 10.1590/1518-8345.2885.3074 30462787 PMC6248737

[28] Hewitt RM, Ploszajski M, Purcell C, Pattinson R, Jones B, Wren GH, et al. A mixed methods systematic review of digital interventions to support the psychological health and well-being of people living with dermatological conditions. Front Med (Lausanne). 2022;9:1024879. doi: 10.3389/fmed.2022.1024879 36405626 PMC9669071

[29] Page MJ, McKenzie JE, Bossuyt PM, Boutron I, Hoffmann TC, Mulrow CD, et al. The PRISMA 2020 statement: an updated guideline for reporting systematic reviews. BMJ. 2021;372:n71. doi: 10.1136/bmj.n71 33782057 PMC8005924

[30] Ouzzani M, Hammady H, Fedorowicz Z, Elmagarmid A. Rayyan-a web and mobile app for systematic reviews. Syst Rev. 2016;5(1):210. doi: 10.1186/s13643-016-0384-4 27919275 PMC5139140

[31] Hong QN, Fàbregues S, Bartlett G, et al. The Mixed Methods Appraisal Tool (MMAT) version 2018 for information professionals and researchers. Educ Inf. 2018;34:285–91.

[32] Wu F, Laza-Cagigas R, Rampal T. Understanding patients’ experiences and perspectives of tele-prehabilitation: a qualitative study to inform service design and delivery. Clin Pract. 2022;12(4):640–52. doi: 10.3390/clinpract12040067 36005070 PMC9406597

[33] Bruns ERJ, Argillander TE, Schuijt HJ, van Duijvendijk P, van der Zaag ES, Wassenaar EB, et al. Fit4SurgeryTV at-home prehabilitation for frail older patients planned for colorectal cancer surgery: a pilot study. Am J Phys Med Rehabil. 2019;98(5):399–406. doi: 10.1097/PHM.0000000000001108 30550454

[34] Bennett E, Clifford T, Cregan F, O’Neill E, Spillane D, Harte K, et al. Experiences implementing a Radiation therapist-led oncology prehabilitation program during COVID-19. Tech Innov Patient Support Radiat Oncol. 2023;29:100226. doi: 10.1016/j.tipsro.2023.100226 38077622 PMC10701593

[35] Piché A, Santa Mina D, Lambert S, Doré I. Assessing real-world implementability of a multimodal group-based tele-prehabilitation program in cancer care: a pragmatic feasibility study. Front Oncol. 2023;13:1271812. doi: 10.3389/fonc.2023.1271812 37965450 PMC10641394

[36] Waller E, Sutton P, Rahman S, Allen J, Saxton J, Aziz O. Prehabilitation with wearables versus standard of care before major abdominal cancer surgery: a randomised controlled pilot study (trial registration: NCT04047524). Surg Endosc. 2022;36(2):1008–17. doi: 10.1007/s00464-021-08365-6 33723969 PMC8758615

[37] MacDonald AM, Chafranskaia A, Lopez CJ, Maganti M, Bernstein LJ, Chang E, et al. CaRE @ home: pilot study of an online multidimensional cancer rehabilitation and exercise program for cancer survivors. J Clin Med. 2020;9(10):3092. doi: 10.3390/jcm9103092 32992759 PMC7600555

[38] Wu F, Rotimi O, Laza-Cagigas R, Rampal T. The feasibility and effects of a telehealth-delivered home-based prehabilitation program for cancer patients during the pandemic. Curr Oncol. 2021;28(3):2248–59. doi: 10.3390/curroncol28030207 34204531 PMC8293185

[39] Gkaintatzi E, Nikolaou CK, Rampal T, Laza-Cagigas R, Zand N, McCrone P. Cost analysis of a digital multimodal cancer prehabilitation. Curr Oncol. 2022;29(12):9305–13. doi: 10.3390/curroncol29120729 36547143 PMC9777147

[40] Phillips C. Mixed methods systematic review using a convergent integrated approach to synthesis and integration. In: Aromataris E, Lockwood C, Porritt K, Pilla B, Jordan Z, editors. JBI manual for evidence synthesis. JBI; 2024. Available from: https://synthesismanual.jbi.global

[41] van Deudekom FJ, Schimberg AS, Kallenberg MH, Slingerland M, van der Velden L-A, Mooijaart SP. Functional and cognitive impairment, social environment, frailty and adverse health outcomes in older patients with head and neck cancer, a systematic review. Oral Oncol. 2017;64:27–36. doi: 10.1016/j.oraloncology.2016.11.013 28024721

[42] Ju M-D, Qin Q, Li M. Whole-process case management effects on mental state and self-care ability in patients with liver cancer. World J Gastrointest Surg. 2024;16(3):833–41. doi: 10.4240/wjgs.v16.i3.833 38577082 PMC10989342

[43] Santa Mina D, Brahmbhatt P, Lopez C, Baima J, Gillis C, Trachtenberg L, et al. The case for prehabilitation prior to breast cancer treatment. PM R. 2017;9(9S2):S305–16. doi: 10.1016/j.pmrj.2017.08.402 28942905

[44] Elkefi S, Trapani D, Ryan S. The role of digital health in supporting cancer patients’ mental health and psychological well-being for a better quality of life: a systematic literature review. Int J Med Inform. 2023;176:105065. doi: 10.1016/j.ijmedinf.2023.105065 37224644

[45] Torous J, Bucci S, Bell IH. The growing field of digital psychiatry: current evidence and the future of apps, social media, chatbots, and virtual reality. World Psychiatry. 2021;20:318–35. 34505369 10.1002/wps.20883 PMC8429349

[46] Murray E, Hekler EB, Andersson G, Collins LM, Doherty A, Hollis C, et al. Evaluating digital health interventions: key questions and approaches. Am J Prev Med. 2016;51(5):843–51. doi: 10.1016/j.amepre.2016.06.008 27745684 PMC5324832

[47] Chen Y-Y, Guan B-S, Li Z-K, Li X-Y. Effect of telehealth intervention on breast cancer patients’ quality of life and psychological outcomes: a meta-analysis. J Telemed Telecare. 2018;24(3):157–67. doi: 10.1177/1357633X16686777 28081664

[48] Corbett T, Cheetham T, Müller AM, Slodkowska-Barabasz J, Wilde L, Krusche A, et al. Exploring cancer survivors’ views of health behaviour change: “Where do you start, where do you stop with everything?” Psychooncology. 2018;27(7):1816–24. doi: 10.1002/pon.4732 29645327

[49] Zhang Y, Flannery M, Zhang Z. Digital health psychosocial intervention in adult patients with cancer and their families: systematic review and meta-analysis. JMIR Cancer. 2024;10:e54722. 10.2196/46116 PMC10877499 38315546

[50] Zion SR, Taub CJ, Heathcote LC. A cognitive behavioral digital therapeutic for anxiety and depression in patients with cancer: a decentralized randomized controlled trial. J Clin Oncol. 2023;41:1507–1507. 10.1200/OP.23.00210 PMC10732510 37862670

[51] Haneczok M, Wojtyna E, M J - A of, et al. The effectiveness of a CBT-based mobile application for the mental state of women with breast cancer. Ann Oncol. 2023;34:S1001.

[52] Lu L, Zhang B, Li W, Li J, Li L. Prevalence and risk factors of psychological distress in patients with early-stage lung cancer during preoperative period: a cross-sectional study. J Clin Nurs. 2025;34(8):3196–205. doi: 10.1111/jocn.17501 39468789

[53] Li J, Khajoueinejad N, Sarfaty E, Yu AT, Troob S, Buseck A, et al. Anxiety and depression are common in surgical oncology patients: results of a prospective cohort study. Surg Oncol Insight. 2024;1(3):100087. doi: 10.1016/j.soi.2024.100087

[54] Schuch FB, Vancampfort D, Firth J, Rosenbaum S, Ward PB, Silva ES, et al. Physical activity and incident depression: a meta-analysis of prospective cohort studies. Am J Psychiatry. 2018;175(7):631–48. doi: 10.1176/appi.ajp.2018.17111194 29690792

[55] Stubbs B, Vancampfort D, Rosenbaum S, Firth J, Cosco T, Veronese N, et al. An examination of the anxiolytic effects of exercise for people with anxiety and stress-related disorders: a meta-analysis. Psychiatry Res. 2017;249:102–8. doi: 10.1016/j.psychres.2016.12.020 28088704

[56] Rose G, Bruni P, Wingood M, Kallmi S, Finer E, Bamonti P. Efficacy of combined therapeutic exercise and psychological interventions on disability outcomes: a scoping review. Innov Aging. 2023;7(Supplement_1):759–60. doi: 10.1093/geroni/igad104.2456

[57] Annesi JJ. Supported exercise improves controlled eating and weight through its effects on psychosocial factors: extending a systematic research program toward treatment development. Perm J. 2012;16(1):7–18. doi: 10.7812/11-136 22529754 PMC3327117

[58] Linardon J, Fuller-Tyszkiewicz M. Attrition and adherence in smartphone-delivered interventions for mental health problems: a systematic and meta-analytic review. J Consult Clin Psychol. 2020;88(1):1–13. doi: 10.1037/ccp0000459 31697093

[59] Mavros MN, Athanasiou S, Gkegkes ID, Polyzos KA, Peppas G, Falagas ME. Do psychological variables affect early surgical recovery? PLoS One. 2011;6(5):e20306. doi: 10.1371/journal.pone.0020306 21633506 PMC3102096

[60] Rosenberger P, Jokl P, Ickovics J. Psychosocial factors and surgical outcomes: an evidence-based literature review. J Am Acad Orthop Surg. 2006;14:397–405. 16822887 10.5435/00124635-200607000-00002

[61] Yang M, Shen Y, Tan L, Li W. Prognostic value of sarcopenia in lung cancer: a systematic review and meta-analysis. Chest. 2019;156(1):101–11. doi: 10.1016/j.chest.2019.04.115 31128115

[62] Shachar SS, Williams GR, Muss HB, Nishijima TF. Prognostic value of sarcopenia in adults with solid tumours: a meta-analysis and systematic review. Eur J Cancer. 2016;57:58–67. doi: 10.1016/j.ejca.2015.12.030 26882087

[63] Baguley BJ, Dalla Via J, Fraser SF, Daly RM, Kiss N. Effectiveness of combined nutrition and exercise interventions on body weight, lean mass, and fat mass in adults diagnosed with cancer: a systematic review and meta-analysis. Nutr Rev. 2023;81(6):625–46. doi: 10.1093/nutrit/nuac079 36206176

[64] Stene GB, Helbostad JL, Balstad TR, Riphagen II, Kaasa S, Oldervoll LM. Effect of physical exercise on muscle mass and strength in cancer patients during treatment--a systematic review. Crit Rev Oncol Hematol. 2013;88(3):573–93. doi: 10.1016/j.critrevonc.2013.07.001 23932804

[65] Yamamoto K, Nagatsuma Y, Fukuda Y, Hirao M, Nishikawa K, Miyamoto A, et al. Effectiveness of a preoperative exercise and nutritional support program for elderly sarcopenic patients with gastric cancer. Gastric Cancer. 2017;20(5):913–8. doi: 10.1007/s10120-016-0683-4 28032232

[66] Gillis C, Buhler K, Bresee L, et al. Effects of nutritional prehabilitation, with and without exercise, on outcomes of patients who undergo colorectal surgery: a systematic review and meta-analysis. Gastroenterology. 2018;155:391–410.e4. 29750973 10.1053/j.gastro.2018.05.012

[67] Burden ST, Hill J, Shaffer JL, Todd C. Nutritional status of preoperative colorectal cancer patients. J Hum Nutr Diet. 2010;23(4):402–7. doi: 10.1111/j.1365-277X.2010.01070.x 20487172

[68] Arends J, Bachmann P, Baracos V, Barthelemy N, Bertz H, Bozzetti F, et al. ESPEN guidelines on nutrition in cancer patients. Clin Nutr. 2017;36(1):11–48. doi: 10.1016/j.clnu.2016.07.015 27637832

[69] Weimann A, Braga M, Carli F, Higashiguchi T, Hübner M, Klek S, et al. ESPEN guideline: clinical nutrition in surgery. Clin Nutr. 2017;36(3):623–50. doi: 10.1016/j.clnu.2017.02.013 28385477

[70] Muscaritoli M, Arends J, Bachmann P, Baracos V, Barthelemy N, Bertz H, et al. ESPEN practical guideline: clinical Nutrition in cancer. Clin Nutr. 2021;40(5):2898–913. doi: 10.1016/j.clnu.2021.02.005 33946039

[71] Zaninotto F, Wynter-Blyth V, Hug A, Halley M, Long L, Rankin M, et al. 1683P Feasibility of implementing a digital prehabilitation service for cancer patients in the NHS. Ann Oncol. 2021;32:S1179. doi: 10.1016/j.annonc.2021.08.1655

[72] Lin Y, Xu X, Liu Y, Alias H, Hu Z, Wong LP. Perception and acceptance of telemedicine use in health care among the general public in china: web-based cross-sectional survey. J Med Internet Res. 2024;26:e53497. doi: 10.2196/53497 39012687 PMC11289571

[73] Granström E, Wannheden C, Brommels M, Hvitfeldt H, Nyström ME. Digital tools as promoters for person-centered care practices in chronic care? Healthcare professionals’ experiences from rheumatology care. BMC Health Serv Res. 2020;20(1):1108. doi: 10.1186/s12913-020-05945-5 33261602 PMC7709268

[74] Zhang Y, Xu P, Sun Q. Factors influencing the e-health literacy in cancer patients: a systematic review. J Cancer Surviv. 2023. 2022;17:425–40. 36190672 10.1007/s11764-022-01260-6 PMC9527376

[75] Arias López MDP, Ong BA, Borrat Frigola X, Fernández AL, Hicklent RS, Obeles AJT, et al. Digital literacy as a new determinant of health: a scoping review. PLOS Digit Health. 2023;2(10):e0000279. doi: 10.1371/journal.pdig.0000279 37824584 PMC10569540

[76] Juraschek SP, Plante TB, Charleston J. Use of online recruitment strategies in a randomized trial of cancer survivors. Clin Trials. 2018;15:130–8. 29361843 10.1177/1740774517745829 PMC5891380

[77] Kemp E, Trigg J, Beatty L, Christensen C, Dhillon HM, Maeder A, et al. Health literacy, digital health literacy and the implementation of digital health technologies in cancer care: the need for a strategic approach. Health Promot J Austr. 2021;32 Suppl 1:104–14. doi: 10.1002/hpja.387 32681656

[78] Crafoord M-T, Fjell M, Sundberg K, Nilsson M, Langius-Eklöf A. Engagement in an interactive app for symptom self-management during treatment in patients with breast or prostate cancer: mixed methods study. J Med Internet Res. 2020;22(8):e17058. doi: 10.2196/17058 32663140 PMC7445604

[79] Krebs P, Prochaska JO, Rossi JS. A meta-analysis of computer-tailored interventions for health behavior change. Prev Med. 2010. 2010;51:214–21. 20558196 10.1016/j.ypmed.2010.06.004 PMC2939185

[80] Lustria MLA, Noar SM, Cortese J, Van Stee SK, Glueckauf RL, Lee J. A meta-analysis of web-delivered tailored health behavior change interventions. J Health Commun. 2013;18(9):1039–69. doi: 10.1080/10810730.2013.768727 23750972

[81] Thelwell MJ, Myers A, Humphreys L. O.3.2-1 Evaluation of the Active Together multi-modal cancer rehabilitation service embedded within clinical care. Eur J Public Health. 2023;33.

[82] Little P, Bradbury K, Stuart B, Barnett J, Krusche A, Steele M, et al. Digital intervention (Renewed) to support symptom management, wellbeing, and quality of life among cancer survivors in primary care: a randomised controlled trial. Br J Gen Pract. 2025;75(754): e357–e365. doi: 10.3399/BJGP.2023.0262 10.3399/BJGP.2023.0262 PMC11755581 38164562

[83] Anieto EM, Dall PM, Abaraogu U, Chastin S, Anieto I. The effectiveness of co-created lifestyle interventions in improving health behaviour, physical and mental health in adults with non-communicable diseases: a systematic review with meta-analysis. Public Health. 2025;248 1:105929. doi: 10.1016/j.puhe.2025.105929 40845709 10.1016/j.puhe.2025.105929

[84] Crowe S, Adebajo A, Esmael H, Denegri S, Martin A, McAlister B, et al. 'All hands on deck', working together to develop UK standards for public involvement in research. Res Involv Engagem. 2020;6 1:53. doi: 10.1186/s40900-020-00229-y 32974049 10.1186/s40900-020-00229-y PMC7493420

[85] Staniszewska S, Brett J, Simera I, Seers K, Mockford C, Goodlad S, et al. GRIPP2 reporting checklists: tools to improve reporting of patient and public involvement in research. BMJ. 2017;358:j3453. doi: 10.1136/bmj.j3453 28768629 PMC5539518

[86] Linedale EC, Bills E, Dimopoulos A, Yeoh J, Nolan M, Hume V, et al. Development of a feasible and acceptable digital prehabilitation pathway to improve elective surgical outcomes. Front Digit Health. 2023;5 1:1054894. doi: 10.3389/fdgth.2023.1054894 36845335 10.3389/fdgth.2023.1054894 PMC9947781

[87] Ferri Sanz M, Vallina Acha B, Ferrando García M. Co-design for people-centred care digital solutions: a literature review. Int J Integr Care. 2021;21(2):16. doi: 10.5334/ijic.5573 33981193 PMC8086727

[88] Chunara R, Zhao Y, Chen J, Lawrence K, Testa PA, Nov O, et al. Telemedicine and healthcare disparities: a cohort study in a large healthcare system in New York City during COVID-19. J Am Med Inform Assoc. 2021;28(1):33–41. doi: 10.1093/jamia/ocaa217 32866264 PMC7499631

[89] Crawford A, Serhal E. Digital health equity and COVID-19: the innovation curve cannot reinforce the social gradient of health. J Med Internet Res. 2020;22:e19361. 10.2196/19361 PMC7268667 32452816

[90] Federal Communications Commission (FCC). Broadband deployment report. Washington (DC): FCC; 2024. p. 1–70. Available from: https://docs.fcc.gov/public/attachments/DOC-405487A1.pdf

[91] European Commission, Directorate-General for Communications Networks, Content and Technology. Broadband coverage in Europe 2023 – Mapping progress towards the coverage objectives of the digital decade – Final report. Luxembourg: Publications Office of the European Union; 2024.

[92] Rendle KA, Tan ASL, Spring B. A framework for integrating telehealth equitably across the cancer care continuum. JNCI Monogr. 2024;2024:92–9. 10.1093/jncimonographs/lgae021 PMC11207920 38924790

[93] Baumann AA, Cabassa LJ. Reframing implementation science to address inequities in healthcare delivery. BMC Health Serv Res. 2020;20(1):190. doi: 10.1186/s12913-020-4975-3 32164706 PMC7069050

[94] Wallerstein N. Engage for equity: advancing the fields of community-based participatory research and community-engaged research in community psychology and the social sciences. Am J Community Psychol. 2021;67:251–5. 34237169 10.1002/ajcp.12530

[95] Shelton RC, Adsul P, Oh A. Recommendations for addressing structural racism in implementation science: a call to the field. Ethn Dis. 2021;31(Suppl 1):357–64. doi: 10.18865/ed.31.S1.357 34045837 PMC8143847

