# Supplementary material for: Feasibility, acceptability and effectiveness of digitally delivered multimodal prehabilitation for cancer patients: A mixed-methods systematic review
Source: PLOS Digit Health. 2026 Mar 2;5(3):e0001258. doi: 10.1371/journal.pdig.0001258 (PMC12952641; doi:10.1371/journal.pdig.0001258)
Supplement: S1 Appendix — (DOCX) [file pdig.0001258.s002.docx]

Supplementary Document

**Feasibility, Acceptability and Effectiveness of Digitally Delivered Multimodal Prehabilitation for Cancer Patients: A Mixed-Methods Systematic Review**

Jeremiah Oyedemi^a^, Simon Dunne^b^, Louise Brennan^c,d^, Laura Coffey^a^

^a^Department of Psychology, Maynooth University, Maynooth, Co. Kildare, Ireland

^b^School of Psychology, Dublin City University, Glasnevin, Dublin, Ireland

^c^Mercer's Institute for Successful Ageing, St James's Hospital, Dublin, Ireland

^d^Discipline of Physiotherapy, School of Medicine, Trinty College Dublin, Dublin, Ireland

**Corresponding author:** Dr Laura Coffey, Department of Psychology, Maynooth University, Maynooth, Co. Kildare, Ireland (tel: +353 1 4747625, e-mail: [laura.coffey@mu.ie](mailto:laura.coffey@mu.ie))

**Table 1: Search Queries**

| Database: Web of science | | | | | | | |  |
| --- | --- | --- | --- | --- | --- | --- | --- | --- |
| Date of search: 24/02/2024 | | | | | | | |  |
| **Search #** | **Concept/Explanation** | **Search Terms/Strategy** | | | **# of Results** | | |  |
| #1 | Cancer | **oncolog* OR “cancer*” OR malignan* OR neoplas* OR tumor OR leukemia OR sarcoma OR carcinoma* OR tumour*** | | | 3.5million | | | |
| #2 | Prehabilitation | “prehab*” OR “pre-hab*” OR "e-Prehab*" OR "E Prehab*" OR "Early Therapy" OR “pre-operative exercise*” OR “preoperative exercise*” OR “pre-operative rehabilitation” OR “preoperative rehabilitation” OR “peri-operative exercise*” OR “perioperative exercise*” OR “peri-operative rehabilitation” OR “perioperative rehabilitation” OR "preoperative period" OR "preoperative care" OR (("presurg*" OR "pre-surg*" OR "pretreat*" OR "pre-treat*" OR “pre hab*” OR prehab* OR pre-op* OR preop* OR peri-op* OR periop*) NEAR/10 (exercise OR physical OR physio* OR diet* OR food OR nutrition* OR psycho*)) | | | 21,090 | | | |
| #3 | Technology | "Telerehab*" OR "Tele-rehab*" OR "Exergam*" OR "Telemedicine" OR "Tele-medicine" OR "Wearable*" OR "Internet of Things" OR "IOT" OR “Internet*” OR "Patient Portals" OR “Virtual*” OR "Smart*" OR "Online*" OR "Digital*" OR "technolog*" OR "mobile*" OR "ehealth*" OR "e-health*" OR "Health Tech*" OR "Sensor*" OR "Telehealth*" OR "Tele-health*" OR "Telecare" OR "Tele-care" OR "Teletherapy" OR "Tele-therapy" OR "M-health" OR "Mhealth" OR "Mobile*" OR "Use of technolog*" OR "remote*" OR “Teleconsult*” OR “Tele-consult*” OR “Software*” OR “Portable Electronic App*” OR “Portable Software App*” OR “Portable Electronic App*” OR “Portable Electronic Application*” | | | 5.8million | | | |
|  |  | #1 AND #2 AND #3 | | | 420 | | | |
|  | Delimiter: | English language  Year | | | 397 | | |  |
|  |  |  | | |  | | |  |
| Database: Embase | | | | | | | |  |
| Date of search: 24/02/2024 | | | | | | | |  |
| **Search #** | **Concept/Explanation** | | | **Search Terms/Strategy** | | | **# of Results** |  |
| #1 | Cancer | | | **oncolog* OR “cancer*” OR malignan* OR neoplas* OR tumor OR leukemia OR sarcoma OR carcinoma* OR tumour*** | | | 6million |  |
| #2 | Prehabilitation | | | “prehab*” OR “pre-hab*” OR "e-Prehab*" OR "E Prehab*" OR "Early Therapy" OR “pre-operative exercise*” OR “preoperative exercise*” OR “pre-operative rehabilitation” OR “preoperative rehabilitation” OR “peri-operative exercise*” OR “perioperative exercise*” OR “peri-operative rehabilitation” OR “perioperative rehabilitation” OR "preoperative period" OR "preoperative care" OR (("presurg*" OR "pre-surg*" OR "pretreat*" OR "pre-treat*" OR “pre hab*” OR prehab* OR pre-op* OR preop* OR peri-op* OR periop*) NEAR/10 (exercise OR physical OR physio* OR diet* OR food OR nutrition* OR psycho*)) | | | 39,725 |  |
| #3 | Technology | | | "Telerehab*" OR "Tele-rehab*" OR "Exergam*" OR "Telemedicine" OR "Tele-medicine" OR "Wearable*" OR "Internet of Things" OR "IOT" OR “Internet*” OR "Patient Portals" OR “Virtual*” OR "Smart*" OR "Online*" OR "Digital*" OR "technolog*" OR "mobile*" OR "ehealth*" OR "e-health*" OR "Health Tech*" OR "Sensor*" OR "Telehealth*" OR "Tele-health*" OR "Telecare" OR "Tele-care" OR "Teletherapy" OR "Tele-therapy" OR "M-health" OR "Mhealth" OR "Mobile*" OR "Use of technolog*" OR "remote*" OR “Teleconsult*” OR “Tele-consult*” OR “Software*” OR “Portable Electronic App*” OR “Portable Software App*” OR “Portable Electronic App*” OR “Portable Electronic Application*” | | | 3million |  |
| #4 |  | | | #1 AND #2 AND #3 | | | 600 |  |
|  | Delimiter: | | | English language  Year | | | 579 |  |
|  |  | | |  | | |  |  |
| Database: Scopus | | | | | | | |  |
| Date of search: 24/02/2024 | | | | | | | |  |
| **Search #** | **Concept/Explanation** | | | **Search Terms/Strategy** | | **# of Results** | |  |
| #1 | Cancer | | | **oncolog* OR “cancer*” OR malignan* OR neoplas* OR tumor OR leukemia OR sarcoma OR carcinoma* OR tumour*** | | 5.36million | |  |
| #2 | Prehabilitation | | | “prehab*” OR “pre-hab*” OR "e-Prehab*" OR "E Prehab*" OR "Early Therapy" OR “pre-operative exercise*” OR “preoperative exercise*” OR “pre-operative rehabilitation” OR “preoperative rehabilitation” OR “peri-operative exercise*” OR “perioperative exercise*” OR “peri-operative rehabilitation” OR “perioperative rehabilitation” OR "preoperative period" OR "preoperative care" OR (("presurg*" OR "pre-surg*" OR "pretreat*" OR "pre-treat*" OR “pre hab*” OR prehab* OR pre-op* OR preop* OR peri-op* OR periop*) W/10 (exercise OR physical OR physio* OR diet* OR food OR nutrition* OR psycho*)) | | 39,463 | |  |
| #3 | Technology | | | "Telerehab*" OR "Tele-rehab*" OR "Exergam*" OR "Telemedicine" OR "Tele-medicine" OR "Wearable*" OR "Internet of Things" OR "IOT" OR “Internet*” OR "Patient Portals" OR “Virtual*” OR "Smart*" OR "Online*" OR "Digital*" OR "technolog*" OR "mobile*" OR "ehealth*" OR "e-health*" OR "Health Tech*" OR "Sensor*" OR "Telehealth*" OR "Tele-health*" OR "Telecare" OR "Tele-care" OR "Teletherapy" OR "Tele-therapy" OR "M-health" OR "Mhealth" OR "Mobile*" OR "Use of technolog*" OR "remote*" OR “Teleconsult*” OR “Tele-consult*” OR “Software*” OR “Portable Electronic App*” OR “Portable Software App*” OR “Portable Electronic App*” OR “Portable Electronic Application*” | | 11million | |  |
| #4 |  | | | #1 AND #2 AND #3 | | 451 | |  |
|  | Delimiter: | | | English language  Year | | 388 | |  |
|  |  | | |  | |  | |  |
| Database: psychinfo | | | | | | | |  |
| Date of search: 24/02/2024 | | | | | | | |  |
| **Search #** | **Concept/Explanation** | | | **Search Terms/Strategy** | | | **# of Results** |  |
| #1 | Cancer | | | **oncolog* OR “cancer*” OR malignan* OR neoplas* OR tumor OR leukemia OR sarcoma OR carcinoma* OR tumour*** | | | 56,920 |  |
| #2 | Prehabilitation | | | “prehab*” OR “pre-hab*” OR "e-Prehab*" OR "E Prehab*" OR "Early Therapy" OR “pre-operative exercise*” OR “preoperative exercise*” OR “pre-operative rehabilitation” OR “preoperative rehabilitation” OR “peri-operative exercise*” OR “perioperative exercise*” OR “peri-operative rehabilitation” OR “perioperative rehabilitation” OR "preoperative period" OR "preoperative care" OR (("presurg*" OR "pre-surg*" OR "pretreat*" OR "pre-treat*" OR “pre hab*” OR prehab* OR pre-op* OR preop* OR peri-op* OR periop*) NEAR/10 (exercise OR physical OR physio* OR diet* OR food OR nutrition* OR psycho*)) | | | 68 |  |
| #3 | Technology | | | "Telerehab*" OR "Tele-rehab*" OR "Exergam*" OR "Telemedicine" OR "Tele-medicine" OR "Wearable*" OR "Internet of Things" OR "IOT" OR “Internet*” OR "Patient Portals" OR “Virtual*” OR "Smart*" OR "Online*" OR "Digital*" OR "technolog*" OR "mobile*" OR "ehealth*" OR "e-health*" OR "Health Tech*" OR "Sensor*" OR "Telehealth*" OR "Tele-health*" OR "Telecare" OR "Tele-care" OR "Teletherapy" OR "Tele-therapy" OR "M-health" OR "Mhealth" OR "Mobile*" OR "Use of technolog*" OR "remote*" OR “Teleconsult*” OR “Tele-consult*” OR “Software*” OR “Portable Electronic App*” OR “Portable Software App*” OR “Portable Electronic App*” OR “Portable Electronic Application*” | | | 153,535 |  |
| #4 |  | | | #1 AND #2 AND #3 | | | 36 |  |
|  | Delimiter: | | | English language  Year | | | 29 |  |
|  |  | | |  | | |  |  |
| Database: Pubmed | | | | | | | |  |
| Date of search: 24/02/2024 | | | | | | | |  |
| **Search #** | **Concept/Explanation** | | **Search Terms/Strategy** | | | | **# of Results** |  |
| #1 | Breast cancer | | **oncolog*[Title/Abstract] OR "cancer*"[Title/Abstract] OR malignan*[Title/Abstract] OR neoplas*[Title/Abstract] OR tumor[Title/Abstract] OR leukemia[Title/Abstract] OR sarcoma[Title/Abstract] OR carcinoma*[Title/Abstract] OR tumour*[Title/Abstract]** | | | | 4.2 M |  |
| #2 | Prehabilitation | | "prehab*"[Title/Abstract] OR "pre-hab*"[Title/Abstract] OR "e-Prehab*"[Title/Abstract] OR "E Prehab*"[Title/Abstract] OR "Early Therapy"[Title/Abstract] OR "pre-operative exercise*"[Title/Abstract] OR "preoperative exercise"[MeSH Terms] OR "pre-operative rehabilitation" [Title/Abstract] OR "preoperative rehabilitation" [Title/Abstract] OR "peri-operative exercise*"[Title/Abstract] OR "preoperative period"[MeSH Terms] OR "preoperative care"[MeSH Terms] OR "perioperative exercise*"[Title/Abstract] OR "peri-operative rehabilitation" [Title/Abstract] OR "perioperative rehabilitation" [Title/Abstract] OR (("presurg*"[Title/Abstract] OR "pre-surg*"[Title/Abstract] OR "pretreat*"[Title/Abstract] OR "pre-treat*"[Title/Abstract] OR "pre hab*"[Title/Abstract] OR "prehab*"[Title/Abstract] OR "pre-op*"[Title/Abstract] OR "preop*"[Title/Abstract] OR "peri-op*"[Title/Abstract] OR "periop*"[Title/Abstract] ) NEAR/10 ("exercise" [Title/Abstract] OR "physical" [Title/Abstract] OR "physio*"[Title/Abstract] OR "diet*"[Title/Abstract] OR "food" [Title/Abstract] OR "nutrition*"[Title/Abstract] OR "psycho*"[Title/Abstract] )) | | | | 85,340 |  |
| #3 | Technology | | "telerehab*"[MeSH Terms] OR "Internet of Things"[MeSH Terms] OR "exergaming*"[MeSH Terms] OR "Patient Portals"[MeSH Terms] OR "Virtual Reality"[MeSH Terms] OR "smartphone*"[MeSH Terms] OR "internet*"[MeSH Terms] OR "Digital Technology"[MeSH Terms] OR "Telemedicine"[MeSH Terms] OR "Telemedicine"[Title/Abstract] OR "Tele-medicine"[Title/Abstract] OR "wearable*"[Title/Abstract] OR "tele rehab*"[Title/Abstract] OR "IOT"[Title/Abstract] OR "online*"[Title/Abstract] OR "technolog*"[Title/Abstract] OR "mobile*"[Title/Abstract] OR "ehealth*"[Title/Abstract] OR "e health*"[Title/Abstract] OR "health tech*"[Title/Abstract] OR "sensor*"[Title/Abstract] OR "telehealth*"[Title/Abstract] OR "tele health*"[Title/Abstract] OR "Telecare"[Title/Abstract] OR "Tele-care"[Title/Abstract] OR "Teletherapy"[Title/Abstract] OR "Tele-therapy"[Title/Abstract] OR "M-health"[Title/Abstract] OR "Mhealth"[Title/Abstract] OR "mobile*"[Title/Abstract] OR "remote*"[Title/Abstract] OR "teleconsult*"[Title/Abstract] OR "tele consult*"[Title/Abstract] OR "software*"[Title/Abstract] OR "portable electronic app*"[Title/Abstract] OR "portable software app*"[Title/Abstract] OR "portable electronic app*"[Title/Abstract] OR "portable electronic application*"[Title/Abstract] OR “Wed*” OR “digital”* [Title/Abstract] OR home-based | | | | 1.89M |  |
| #4 |  | | #1 AND #2 AND #3 | | | | 696 |  |
|  | Delimiter: | | English language | | | | 603 |  |
|  |  | |  | | | |  |  |

Table 2: Quality Appraisal

**Quality Appraisal using Mixed Methods Appraisal Tool (MMAT: citation needed)**

|  | Study | Q1 | Q2 | Q3 | Q4 | Q5 | Total Score |
| --- | --- | --- | --- | --- | --- | --- | --- |
| Non-randomized controlled trials | Moorthy et al., 2023 | N | Y | N | N | N | 20% |
|  | Piche et al., 2023 | N | Y | Y | N | Y | 60% |
|  | Steffens et al., 2023 | N | Y | N | N | UC | 40% |
|  | Wu et al., 2021 | Y | Y | N | N | UC | 40% |
|  | Bruns et al., 2018 | Y | Y | Y | N | Y | 80% |
|  | Bennett et al. 2023 | Y | Y | Y | N | Y | 80% |
|  | Gkaintatzi et al., 2022 | UC | Y | N | N | Y | 40% |
|  | Waterland et al., 2021 | Y | N | Y | N | Y | 60% |
|  | Li et al., 2024 | UC | Y | N | UC | Y | 40% |
| Qualitative study | Wu et al., 2022 | Y | Y | Y | Y | Y | 100% |
| Mixed Methods | MacDonald et al., 2020 | N | Y | Y | N | Y | 60% |
| Randomized controlled trials | Waller et al., 2022 | Y | Y | Y | N | N | 60% |

| N: no; Y: yes; UC: unclear |
| --- |
| MMATchecklist for Non-randomized controlled trials: Q1: Are the participants representative of the target population? Q2: Are measurements appropriate regarding both the outcome and intervention (or exposure)? Q3: Are there complete outcome data? Q4: Are the confounders accounted for in the design and analysis? Q5: During the study period, is the intervention administered (or exposure occurred) as intended? |
| Prisma checklist for Qualitative study: Q1: Is the qualitative approach appropriate to answer the research question? Q2: Are the qualitative data collection methods adequate to address the research question? Q3: Are the findings adequately derived from the data? Q4: Is the interpretation of results sufficiently substantiated by data? Q5: Is there coherence between qualitative data sources, collection, analysis and interpretation? |
| Prisma checklist for Mixed Methods: Q1: Is there an adequate rationale for using a mixed methods design to address the research question? Q2: Are the different components of the study effectively integrated to answer the research question? Q3: Are the outputs of the integration of qualitative and quantitative components adequately interpreted? Q4: Are divergences and inconsistencies between quantitative and qualitative results adequately addressed? Q5: Do the different components of the study adhere to the quality criteria of each tradition of the methods involved? |
| Prisma checklist for Randomized controlled trials: Q1: Is randomization appropriately performed? Q2; Are the groups comparable at baseline? Q3: Are there complete outcome data? Q4: Are outcome assessors blinded to the intervention provided? Q5: Did the participants adhere to the assigned intervention? |

Qualitative Findings:

Theme 1: Accessibility and Convenience of Remote Delivery

A prominent theme across multiple studies was that tele-prehabilitation improved accessibility and convenience for participants. Wu et al. (2022) identified "convenience of tele-prehabilitation" as a major theme, with participants appreciating the flexibility to incorporate exercise into daily routines around work or personal commitments. Participants valued avoiding hospital travel due to cost constraints (public transport, parking) or time limitations. One participant noted: "Having prehabilitation outside of the hospital setting made things easier. I wasn't feeling good with the pain and couldn't travel too far. Could also do it in my own time" (Wu et al., 2022).

The remote format widened accessibility beyond geographical catchment areas and accommodated participants with physical limitations that would have restricted attendance at in-centre sessions—including pain, poor mobility, or bowel dysfunction. MacDonald et al. (2020) found participants enjoyed "being able to take part in the program from home rather than having to travel to the hospital," with one participant stating: "I could certainly have gotten on the subway and made the trek downtown but I just like the convenience and for me it's just more practical to be able to do them at home."

Additionally, psychological benefits of remote delivery emerged. Wu et al. (2022) reported that participants with anxiety found hospital visits and clinical interactions heightened stress levels, as hospital appointments were "associated with days with low mood...Never quite sure what the hospital agenda is." Similarly, Waterland et al. (2021) found that 77% of participants preferred online sessions over hospital-based education, appreciating not having to travel and the ability to include family members.

Theme 2: Technical and Digital Literacy Challenges

Despite the benefits of remote delivery, technical challenges and digital literacy barriers emerged as significant concerns. Steffens et al. (2023) found that 21.4% of participants needed technical support to navigate the preoperative online program, with barriers including "poor preoperative health," "lack of motivation," and "lack of personal encouragement." Participants highlighted that "simplicity" and perceived "benefits" were key facilitators for uptake.

Wu et al. (2022) reported participants mentioned "a lack of digital skills or confidence in using digital devices as a challenge to participating in a home-based prehabilitation programme." Participants also noted that digital resources might be cost-prohibitive for some, "suggesting that not everyone had access to the necessary devices or internet connectivity required to fully engage in the programme."

MacDonald et al. (2020) noted minimal technical issues, with 89% of participants logging into the app, though some participants expressed mixed feelings toward weekly e-modules—finding them too long or lacking sufficient time to complete them despite health coach reminders. Connectivity issues were also reported; Moorthy et al. (2023) experienced initial connectivity issues with wearables, with data reliability improving from 25% to 86% over time.

Theme 3: The Critical Role of Human Connection and Support

A recurring theme was the essential value of human connection within otherwise digital interventions. MacDonald et al. (2020) found that health coaching calls were identified as "a valuable program component that encouraged accountability and provided an appreciated human touch element and support during a time when they often felt alone." Participants stated: "I always enjoyed the check-in because it was an opportunity to share insights or ask questions and clarify things...I think it's really extremely extremely valuable" (MacDonald et al., 2020).

Wu et al. (2022) identified "patient-professional relationship" as a key motivator, with regular one-to-one interactions enabling participants to build trust and rapport. Weekly or biweekly phone calls served as opportunities for goal-setting and positive reinforcement: "I had one-to-one contact with [prehabilitation professional], so I was able to speak to him and kind of build up a rapport...I think having been able to have that continuity with the support I had was very important to me."

Conversely, the absence of peer support was identified as a missed opportunity. Wu et al. (2022) reported that participants expressed feelings of isolation and sought advice from online patient forums, wanting to connect with other cancer patients with similar experiences. Participants noted: "I would have liked to have contact with other people that are going through cancer or have had gone through cancer...You don't want to burden your friends or your family with what you're going through. So perhaps having like a face-to-face support group might have been helpful or perhaps group sessions."

Similarly, MacDonald et al. (2020) found some participants appreciated having a non-group option due to privacy concerns, while others may have benefited from shared experiences.

Theme 4: Personalization and Self-Efficacy Development

Participants valued personalized interventions that developed their self-efficacy and sense of control. Wu et al. (2022) identified "personalised service" and "self-efficacy" as key capability themes. Participants received tailored advice according to their specific treatments and symptoms, which they felt was not readily available outside prehabilitation programs. Exercise physiologists collaborated with patients to design personalized plans and set achievable goals, bringing feelings of success and empowerment: "Now I have a feeling of control over my body...I don't want cancer to define me" (Wu et al., 2022).

The multimodal nature of interventions was also valued. Wu et al. (2022) found that participants perceived their abilities to adhere required a holistic approach, with psychological counselling being a key component: "I see them as all together. Without the mental part, it is difficult to have the motivation to keep doing the exercises and keep the nutrition side of things going...If you take one away, it doesn't work" (Wu et al., 2022).

MacDonald et al. (2020) reported participants spoke about benefits including their ability to self-manage emotions and exercise, with many gaining "a new appreciation for exercise" and learning "how to realistically fit exercise into their daily and weekly schedules." Some participants felt better able to accept their diagnosis and manage emotions surrounding it as a result of the program.

Steffens et al. (2023) found that most participants (76%) believed the program would benefit their recovery, with 86.2% feeling confident and 89.7% feeling safe performing the program at home. Notably, 93.1% would complete the program if their surgeon recommended it, highlighting the influence of clinical endorsement.

Motivation for participation was strongly linked to immediate perioperative benefit rather than long-term health gains. Wu et al. (2022) found all patients reported favorable attitudes toward participating for immediate recovery benefits: "You've got to keep your body physically fit...So, if you do have to have aggressive treatment or surgery, then you're more likely to recover from it." Support from family and friends also served as a strong motivator, with household members often engaging in the program alongside participants.

Summary: Qualitative findings reveal that digital multimodal prehabilitation offers valued accessibility and convenience benefits, particularly for patients with physical limitations, geographical barriers, or hospital anxiety. However, technical literacy challenges and the need for human connection—both from professionals and peers—remain important considerations. Personalized, multimodal approaches that develop self-efficacy and are endorsed by treating clinicians are most likely to engage participants, particularly when framed around immediate perioperative benefits rather than long-term health promotion.
